# Summary of over Fifty Years with Brain-Computer Interfaces—A Review

**DOI:** 10.3390/brainsci11010043

**Published:** 2021-01-03

**Authors:** Aleksandra Kawala-Sterniuk, Natalia Browarska, Amir Al-Bakri, Mariusz Pelc, Jaroslaw Zygarlicki, Michaela Sidikova, Radek Martinek, Edward Jacek Gorzelanczyk

**Affiliations:** 1 Faculty of Electrical Engineering, Automatic Control and Informatics, Opole University of Technology, 45-758 Opole, Poland; natalia.browarska@gmail.com (N.B.); m.pelc@greenwich.ac.uk (M.P.); j.zygarlicki@po.edu.pl (J.Z.); 2 Department of Biomedical Engineering, College of Engineering, University of Babylon, 51001 Babylon, Iraq; amir.albakri80@gmail.com; 3 Department of Computing and Information Systems, University of Greenwich, London SE10 9LS, UK; 4 Department of Cybernetics and Biomedical Engineering, VSB-Technical University Ostrava—FEECS, 708 00 Ostrava-Poruba, Czech Republic; michaela.sidikova@vsb.cz (M.S.); radek.martinek@vsb.cz (R.M.); 5 Department of Theoretical Basis of BioMedical Sciences and Medical Informatics, Nicolaus Copernicus University, Collegium Medicum, 85-067 Bydgoszcz, Poland; medsystem@medsystem.com.pl; 6 Institute of Philosophy, Kazimierz Wielki University, 85-092 Bydgoszcz, Poland; 7 Babinski Specialist Psychiatric Healthcare Center, Outpatient Addiction Treatment, 91-229 Lodz, Poland; 8 The Society for the Substitution Treatment of Addiction “Medically Assisted Recovery”, 85-791 Bydgoszcz, Poland

**Keywords:** signal processing methods, Brain-Computer Interfaces, neuro-imaging, electroencephalography, electrocorticography

## Abstract

Over the last few decades, the Brain-Computer Interfaces have been gradually making their way to the epicenter of scientific interest. Many scientists from all around the world have contributed to the state of the art in this scientific domain by developing numerous tools and methods for brain signal acquisition and processing. Such a spectacular progress would not be achievable without accompanying technological development to equip the researchers with the proper devices providing what is absolutely necessary for any kind of discovery as the core of every analysis: the data reflecting the brain activity. The common effort has resulted in pushing the whole domain to the point where the communication between a human being and the external world through BCI interfaces is no longer science fiction but nowadays reality. In this work we present the most relevant aspects of the BCIs and all the milestones that have been made over nearly 50-year history of this research domain. We mention people who were pioneers in this area as well as we highlight all the technological and methodological advances that have transformed something available and understandable by a very few into something that has a potential to be a breathtaking change for so many. Aiming to fully understand how the human brain works is a very ambitious goal and it will surely take time to succeed. However, even that fraction of what has already been determined is sufficient e.g., to allow impaired people to regain control on their lives and significantly improve its quality. The more is discovered in this domain, the more benefit for all of us this can potentially bring.

## 1. Introduction

Rapid technological development, especially during the last 30 years has led to the increased scientific interest in using biomedical data (of various types and for many purposes including communication, movement control, environment control, neurorehabilitation, etc. [1,2,3,4,5,6]. It is also related to the rapid development of the cognitive sciences, which include the following areas [1,7,8,9,10,11]:

- cognitive psychology,
- artificial intelligence,
- neuroscience,
- linguistics,
- anthropology,
- philosophy,
- robotics,
- information technology.

It is also important to mention the occurrence of various Brain-inspired Cognitive Systems (BCSs), which are gradually becoming more and more popular in particular in bio-cybernetics or cognitive science [8]. Due to the fact that the analysis of biomedical signals has become one of the most important diagnostic methods in many research, biological or medicine areas [12].

One of the most important aspects of the biomedical data analysis (which is the predominant authors’ scientific interest), in particular signals, is a desire to develop a perfect, ideal, intuitive, Human-Machine Interface (HMI) [1], to which the Brain-Computer Interfaces belong (it will be presented in detail further in this work) [2,3,13].

As it has been mentioned above—the Human-Machine Interfaces (HMI) are currently playing more and more important role in human lives [1], also because the most modern Human-Machine Interfaces enable direct communication between an external device and a human, so there is no need to use any other control equipment such as mouse or keyboard [3,14].

Such systems can also help some handicapped users to interact with the external environment in a more direct, user-friendly, intuitive way [3,15,16]. Some of the listed below newest HMI systems apply various biomedical signals for control purposes [2,3,11,14,17]:

- electromygraphy—EMG,
- electrooculography—EOG,
- brain signals (electroencephalograhhy—EEG and electrocorticography—ECoG)— (Brain Computer Interfaces).

The HCI systems can be divided into the two following categories [18]:

- neural prostheses, which are a cybernetic alternative for a limb using nerves connected with the muscles;
- Brain-Computer Interfaces (BCI), which detect human decision through electromagnetic pulses directly from the brain.

## 2. Brain-Computer Interfaces

The human brain is claimed to be the most complicated human organ, which could be compared to a very powerful and complex computer, where, until today, no one was able to recreate and simulate successfully its entire structure [10,11,19,20,21].

Recently, a very rapid development of medicine and information technology (and their combination) started the era of the Brain-Computer Interfaces (BCIs), in particular their non-invasive version, based on the electroencephalography (EEG) [2,3].

It is possible to divide the BCIs into invasive and non-invasive systems [2,3,10]. Usually, for either type of BCI information exchanged between the brain and computer (or any other device being part of the BCI system) constitutes data that is being processed in real-time. For that purpose each brain activity has to be measured either directly or indirectly. Direct connection means measurement of electrical activity the brain generates (e.g., EEG), while indirect connection can be performed via [2]:

- blood oxygen measurements,
- functional resonance imaging (fMRI),
- functional infrared spectroscopy (fNIRS), etc…

The development of the BCI systems has advanced from a simple EEG recording into a really efficient Brain-Computer communication [2]. The BCI acquires signals from the brain, then analyzes them and translates into particular commands. Thus, this allows a complete or partial replacement of peripheral devices (computer keyboard, mouse, joystick) to perform an action. BCI from the terminology viewpoint refers to a system, which measures and uses signals acquired from the central nervous system (CNS), which means that e.g., voice-activated and muscle-activated systems could not be called a BCI. According to the definition, a single electroencephalogram (EEG) is not a BCI either because it is only a measurement appliance and does not provide any feedback to its user [22].

**Figure 1:**
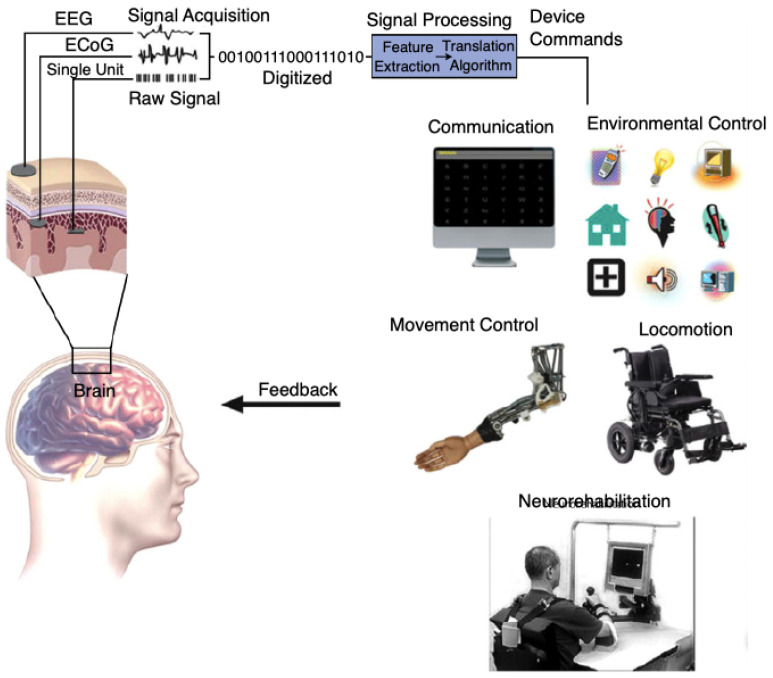
Components of a typical BCI system and its communication methods—simplified scheme.

Obviously, it is equally incorrect to perceive BCIs as mind-reading devices because it is impossible to extract unwilling information from users or their particular thoughts. Thus, the BCIs enable the user to carry out direct action only with their brain activity without muscle engagement [22,23]. In Figure 1 a simplified scheme of a BCI system is presented. The acquired brain signals are initially amplified and then digitized, which simplifies extraction of the particular signal’s characteristics or patterns and then translating them into commands [3]. The obtained result identifies the type of user’s reaction and constitutes one of the mandatory elements of feedback loop.

The brain signals, which represent the overall electrophysiological activity of the brain nerve cells, are obtained from either, the surface of the scalp or directly from the cortical surface. It is a voltage fluctuation generated by the neurons, which can reflect the changes of different human body states [21,24,25].

As majority of the non-invasive BCI systems are based on EEG data, the invasive BCIs are mostly based on signals recorded directly from the brain e.g., electrocorticography [26,27]. The electrocorticogram (ECoG) is an invasive signal reflecting recordings of electrical activity of the brain, which is obtained from macro electrodes (typically 2–3 mm in diameter) placed directly on the cortex surface [26,28,29]. It was developed in 1930 by W. Penfield and H. Jasper as a technique applied for epileptic seizure foci detection [30].

**Figure 2:**
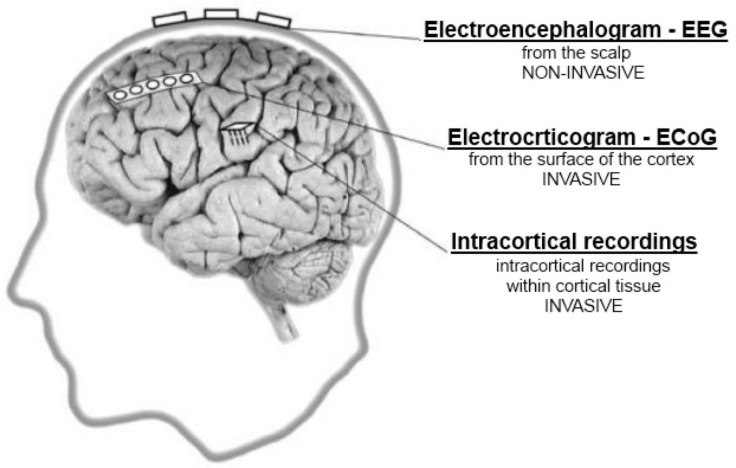
Three different methods for electrical activity of brain recordings [3,22,31].

In Figure 2 three different methods used for the recording of the electrical activity of brain, including one non-invasive (EEG) and two invasive (ECoG and intracortical recordings) are illustrated [3,27,31].

The ECoG recordings provide stronger and better-quality signals than the EEG data, mainly because of their following [22,30]:

- spatial resolution in the millimeters scale,
- frequency bandwidth up to 200 Hz or higher,
- amplitude up to 100 μV,
- reduced sensitivity to movement and myoelectrical artifacts.

The invasive methods have one advantage over the non-invasive methods—the signal is “stronger” and of higher amplitude, which results in more accurate data. The problem is that acquiring these signals is often risky, expensive and requires undertaking major surgery. One of the main disadvantages of the invasive method is that it can only be placed for a very short time and has to be removed as soon as possible as it may cause a tissue damage [4,27,31,32,33].

**Figure 3:**
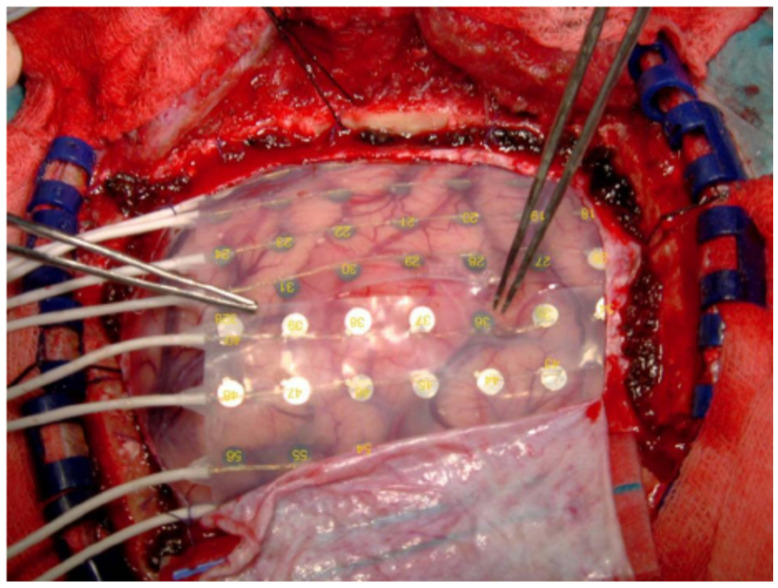
Sample net of electrodes placed on the cortex surface [3,34].

The electrocortical recordings are carried out mostly through electrodes (in form of arrays) placed directly on the cortex surface (see Figure 3)—in this case on the monkeys’ scalp [34,35,36]. As the signal is recorded from the surface of the scalp, the ECoG electrodes are placed on the cortical surface and the electrodes’ spikes enable recording data from local-field potentials (LFPs) [4,22,27].

Although implementation of the invasive recording methods may look painful and it is also very risky (requires longer convalescence), it is important to mention that the brain itself (despite being the most important part of the central nervous system) does not generate any kind of pain [27,31].

The Brain-Computer Interfaces enable direct communication between the human brain and computer. As it was mentioned above—many different kinds of signals can be applied for development of a BCI system [22].

The most popular are non-invasive BCI systems as they do not require any surgical intervention, and is neither their implementation difficult nor risky [3,22]. On the other hand—the invasive BCI systems, despite being risky and requiring surgical intervention, can be much reliable mostly due to the nature of the applied signals (such as inter alia Electrocorticography (ECoG) providing a reliable signal) which can be used to decode movements, vision, and speech [22].

### 2.1. History of Brain-Computer Interfaces

As it was mentioned in the earlier part of this work about non-invasive, EEG-based BCI systems, it is important to start with the short characteristic of the EEG, which was at first recorded by Hans Berger in 1924 and that’s what has led to the identification of the alpha and beta waves [26,37,38,39,40,41]. Hans Berger published in 1930 a paper titled: “Über das Elektrenkephalogramm des Menschen”, where he presented his findings [2,39,42,43].

Discovery of the electroencephalography was a kind of prelude to the development of the BCI systems [2,18,43], however, its history dates back to the 19th century, when the English physicist Richard Caton recorded (as the first ever) animals’ electrical signals and published his results in 1875 in the British Medical Journal [44]. At the same time—two Polish scientists—Napoleon Nikodem Cybulski and Adolf Beck have been working on bioelectrical brain signals recording [45,46].

As the very first experiments were carried out on animals, it is important to mention that in 1913—Vladimir Vladimirovich Prawdicz-Nieminski was the first one to show as many as seven different types of changes in the bioelectrical activity of the brain in animals and to register the alpha and beta frequencies, which he called “electrocerberogram” [46,47].

It is also worth mentioning the enormous contribution of the Russian researcher—Ivan Sechenov, who studied stimulations in the central nervous system and found that the nervous system was reflexive. His student was the creator of the theory of unconditional reflexes—Pavlov [46,48,49].

Despite that Hans Berger was the first one to record the alpha and beta frequencies, he considered them as artifacts [43]. His findings were confirmed in early 1934 by English scientist Lord Adrian and American researcher from the Harvard University—Hallowell Davis [50]. As a useful part of the EEG data these frequencies were only taken into consideration by American pioneers of the electroencephalography (among them was the above mentioned H. Davis): Hallowell Davis, Herbert H. Jasper, Frederic A. Gibbs, William Lennox, and Alfred L. Loomis [17,43,50].

Nowadays, the EEG examination is one of the most popular diagnostics methods of the inter alia below mentioned disorders [26,51,52,53,54,55]:

- Epilepsy,
- Attention Deficit Disorder (ADD),
- Attention-Deficit/Hyperactivity Disorder (ADHD),
- concentration problems,
- Parkinson’s Disease (PD),
- Multiple Sclerosis,
- sleep problems,
- various mental disorders.

Many studies carried out on the EEG signals proved that the specific bands in the signal are closely related to particular functions, and the disproportion in these frequencies is the basis for the diagnosis of certain disorders and diseases [37,51]. Table 1 presents frequency ranges with their short description [26,56,57,58].

The EEG signals can be recorded with the use of various types of electrodes covered with various compounds such as silver chloride or gold. Standard electrode resistance (impedance) is just a few kΩ [26,41,59]. The electrodes placement on the surface of the examined person’s scalp complies with the “10/20” system, where the values “10” and “20” refer to the distance between the measurement points of the arcs running along the three planes [3,59].

Such a thorough history of the electroencephalography (EEG) is being presented in this work as these signals represent electrical activity of the brain and are recorded in a non-invasive way [2,3,26,43,60,61].

It is also important to mention that the EEG signals are considered to be non-deterministic and that they have no special characteristics like ECG signals, what affects their analysis [58,62,63].

The real research on the BCI began in the 1970s in California (UCLA) with experiments performed on animals to develop a new, direct communication path between external environments (or devices) and the brain [2,18]. In 1973 Jacques Vidal published a paper titled: “Toward Direct Brain-Computer Communications” [64].

The very first tests with the BCI development were carried out on monkeys in 1969 and 1970, while the first attempts with human beings were performed in the 90s. The first full definition of the BCI was provided by Jonathan Wolpaw in 2000 [2,18].

One of the most popular signs describing activity of brain are slow cortical potentials (SCP), which had been used in the past for communication of patients in LIS (Locked-in Syndrome), but now has been replaced with direct current (DC) EEG shifts [2,65], which was described for the first time by Walter et al. in 1964 in [66].

M. Sterman and his colleagues carried out some experiments on cats in the 1970s, where they investigated a rhythmic EEG activity of the frequency ranges 12–15 Hz, which was later labeled as SMR (sensorimotor rhythm) [2,67]. The SMR can be also referred to as μ rhythm [2,68].

The 1970s and 1980s brought growing interests on studies on Event-Related Potentials (ERP) as a brain response to external and internal stimulation [2]. Already in 1988 a paper titled: “Talking off the top of your head” written by L.A. Farwell and E. Donchin on implementation of ERP and as the first ones they introduced a now-famous mode of stimulus presentation, which enabled letter choice [2,69].

In 1991 Wolpaw and his colleagues showed that a cursor on a computer screen can be controlled using brain waves, in particular with the above mentioned μ-rhythm (SMR) [2]. Also in the redbeginning of the 1990s the Pfurtscheller and his team applied the ERD (Event-Related Desynchronization) and ERS (Event-Related Synchronization) as an input signal for controlling a BCI [2,70].

### 2.2. Invasive Brain-Computer Interfaces

In 1998 Philip Kennedy implanted the first invasive BCI into human, in 2003 a first BCI game called “BrainGate” was introduced John Donoghue and in 2004 Matt Nagle (1980–2007) was the first patient with implanted invasive BCI system, who had 3rd category quadriplegia with retained speaking ability. He became quadriplegic following a stabbing in the spine, which unfortunately left him disabled [64,71,72].

In 2006 Leuthardt et al. proved ECoG to be an effective source for control signal in BCI Systems, achieving accuracy between 73% and 100% [27].

As mentioned above—the SCP were applied for the early BCI systems. As it was also possible to change the amplitude and polarity of these potentials voluntarily, it was possible to apply them for clinical application and to allow LIS or ALS (Amyotrophic Lateral Sclerosis) patients to communicate. It has been for the very first time published by N. Birbaumer et al. in 1999 in Nature [73,74].

The 2000s brought a highly increased number of studies and papers about the BCI systems [2]. It is also important to mention the two groundbreaking studies published in 2012 Nature [35,36,75]. The both studies showed how the BCI systems enabled neural arm control and arm movements restoration after paralysis [35]. The first one concerned experiments carried out on monkeys [35,36]. In this study the authors implanted a 100-electrode recording array (Blackrock Microsystems) in the M1 hand area and intramuscular electrodes (during separate surgery) for hand and forearm muscles stimulations and recording. The overall success rate for both animals using the neuroprosthesis was about 80% [36]. The second study was inspired by the first one, but involved two human subjects (58 years old female and 66 years old male), who were tetraplegic and anarthric due to the stroke. The neural signals were recorded with the use of 4×4 mm, 96-channel microelectrode array implanted in the dominant M1 hand area. Both participants were able to move robotic arm, so the applied BCI system restored partially their hand motor ability. The female participant was able to drink on her own for the first time in 14 years [75]. During the new millennium new solutions, which much improved patients’ quality of life have been developed, such as the system applied on Cathy Hutchinson, who was then 58 and unable to move for nearly 14 years. She was able to use a robotic arm for among the others drinking. It significantly improved her quality of life [64,75,76].

Further investigations on tetraplegic patients with implanted invasive BCI system, applied for robotic arm control, were presented in inter alia [11]. In this study two 96-channel intracortical microelectrodes were implanted in the motor cortex area of a 52-year-old female subject. The BCI training lasted 13 weeks with its main aim for controlling an anthropomorphic prosthetic limb with seven degrees of freedom [11].

The first information transfer between two human brains without any kind of intervention of motor or PNS (Peripheral Nervous System) was carried out in 2014 [10].

Another very advanced implementation of an invasive BCI system is the one applied on Tim Hemmes, who was injured in a motorcycle accident. He had an implanted system, which allowed him to recover the tactile sensation of his friend through the BCI system. He was able to “feel” touching another person [21,64].

One of the most interesting studies is the one with a BCI implementation on a non-spastic 24-year-old quadriplegic male. In this case a Utah microelectrode array (Blackrock Microsystems) was implanted in his left primary motor cortex, which was identified through functional magnetic resonance imaging (fMRI) performance while the participant had to mirror videos of hand movements. The patient attended up to three sessions per week for 15 months, where he was trained to use his motor cortical neuronal activity in order to control a custom-built high-resolution neuromuscular electrical stimulator (NMES), which delivered electrical stimulation to his paralysed right forearm muscles. It consisted of an 130-electrodes-array embedded in a custom-made flexible sleeve wrapped around his arm. As a result of this research the participant partially gained wrist and hand function, which gave him some independence in daily life activities. The applied NBS system is invasive, but provides an advantage over existing functional electrical stimulation systems using low-amplitude signals such as EEG or EMG [77].

In [64] a restoration of touch feeling using a BCI system was described, and the hypothesis of the BCI system leveraging sensory incompleteness, enhancing touch events, and simultaneous restoration both, the sense of touch and motor function in a person with a spinal cord injury, was assessed in [78]. Participant of this study was chronically paralyzed and had an intracortical recording array implanted in the primary motor cortex M1. For the study purposes he used his own hand with electrodes wrapped around his forearm (similar to those applied in [77]) [78].

In 2017 Ajiboye et al. [79] described a study with a 53-year-old male with a spinal cord injury, who has implanted two intracortical 96-microelectrode arrays in the hand area of motor cortex and later received a total of 36 implanted percutaneous electrodes in his dominant right side to electrically stimulate his hand, elbow, and shoulder muscles. The participant used a mobile arm support for support against gravity and motorised humeral abduction and adduction. The patient achieved 80–100% success during single-joint movements of the elbow, wrist, hand, and mobile arm support. For other joint movements the participant achieved high success rates either, however, the targets were acquired more slowly. The overall study showed promising results and confirmed the effectiveness of the intracortical BCI systems in impaired people recovery [79,80].

In [81] a study with a 27-year-old male tetraplegic participant was presented. He had implanted a 96-channel micro-electrode array in his left dominant hand and arm area. The patient underwent 80 sessions, where he had to imagine a series of four distinct hand movements, such as e.g., index extension, index flexion, wrist extension, wrist flexion. The obtained results were very promising.

### 2.3. Non-Invasive BCI Systems

The BCI Systems can be divided into the two main categories: invasive and non-invasive [3,82]. Most of the EEG-based BCI systems rely on the listed below paradigms [15,64,70,83,84,85,86,87,88]:

1. ERD—associated with motor imagery (MI),
2. ERP—event-related potentials (P300 and other components),
3. SSVEP—steady-state visual evoked potentials,
4. ASSR—auditory steady-state response,
5. SCP—slow cortical potentials,
6. SMR—sensorimotor oscillations,
7. various hybrid systems (based on more than one input signal).

The most popular and effective are, based on thorough literature review, the ERP-based BCI systems [86,89]. However, the SSVEP systems are considered to be high-speed BCIs as they require less training and are easier to configure [84,85,90]. However, they need gaze control and have been reported as tiring and uncomfortable for users [85]. The P300-based BCIs are very popular as they are the oldest studied paradigms and need relatively little training [84,85,86]. The ASSR-based BCI paradigm is a relatively new BCI, and can be classified as a vision-free BCI model. In contrast to the high-speed, well known SSVEP or the P300 paradigms the participants do not need to move their eyes to enforce desired commands, which could be a good solution for totally paralyzed patients [84].

Based on the thorough background study it is possible to distinguish other than EEG-based non-invasive Brain-Computer Interfaces. However, due to their complex technical requirements, high cost, low portability, limited real-time connection, they are not suitable for daily usage [18,84,91]:

- magnetoencephalography (MEG)—requires large, unhandy equipment;
- functional Magnetic Resonance Imaging (fMRI)—large, expensive, unhandy device, poor temporal resolution;
- near infrared spectroscopy (NIRS)—poor temporal resolution;
- positron emission tomography (PET)—large, expensive, unhandy equipment.

The non-invasive story of the BCIs started much later than the invasive one—in the 90s [92,93,94]. As the BCI definition, which was mentioned above, was at first introduced by Jonathan Wolpaw [92], numerous scientific groups from all over the world became interested in this scientific area such as inter alia scientists from Graz, who developed the Graz-BCI systems [95].

The non-invasive, EEG-based BCI systems have various applications—from gaming to rehabilitation via various external devices control such as inter alia wheelchair, robotic arm or video display [96,97,98,99,100,101,102,103]. The main aim of development of non-invasive systems was the need for finding an alternative way of control and communication for handicapped users, as those fully impaired or paralysed are unable to use conventional assisting devices due to the necessity of using some degree of muscle functions [3,99,101].

Rapid development of the digital signal processing technologies enables more efficient analysis of the EEG signals, which led to the further development of the EEG-based BCIs [94,99].

McFarland et al. in 1997 ([99]) proposed a 64-EEG-channel system, with real-time spatial filtering and spectral analyses performance. The user was able to control a video display using his “thoughts” only. The data was processed on-line, but stored for further off-line analysis, which allowed the whole processing evaluation. Only one year later, in 1998—Miner et al. ([100] presented a similar study, where four adults, including one suffering ALS learned to use μ- and β-rhythm-based BCI in order to move cursor on a video screen. Their efficiency was between 93% and 99%. Further investigations, such as those from Pfurtscheller et al. ([104] proved that it was possible to use the EEG data for the cursor control purposes.

At the beginning the EEG-based BCIs applied the CSP-based algorithms, which were at first introduced by the Graz BCI group. They operated on a 27-channel EEG [105].

Desire for further BCI development for patients with severe motor disabilities has brought the BCI2000 project by the scientists from the Wadsworth Center, which unlike other BCI systems, is not designed for one purpose only, but is rather a type of a general-purpose system. The BCI2000 allows to combine various brain signals, various signal processing methods and operating protocols. It is a free of charge, well documented project, recommended for among the others educational purposes [101,105,106]. Their BCIs work based on the SMRs or P300s [105,106].

The groundbreaking milestone in the development of the BCI systems are, as it was already mentioned above, the researchers from Graz. In 2003 they proposed a cue-based system, which applied imagery motor action and translated it into control command enabling to control a virtual keyboard or a hand orthosis [107].

Based on the experience with the off-line results del Millan et al. suggested in 2002 ([108]) to use local neural classifier, which was based on quadratic discriminant analysis. After few days of training only, the study participants were able to reach correct command recognition of 75% [105,108]. This system was later applied for the purpose of a motorized wheelchair and a virtual keyboard control [102,105].

A huge impact on the development of this scientific area had also scientists from Berlin, who developed a project called Berlin-BCI (BBCI). Their solution was efficient, however required a very long (over 20 min) calibration. Their BCI was based on the SMR and required little or no training, which makes it more flexible [105].

The main aim for the development of the BCI system was the need for communication enabling for handicapped people, however, some interesting projects, where the systems were destined for pure entertainment only, have also to be mentioned, such as the one presented in [98], where the EEG signals were used for playing pinball.

One of the interesting projects is the one developed by del Millan et al. in 2008, where the participants were able to control a wheelchair. The authors proposed asynchronous EEG-based BCI, which allowed operating of a wheelchair for a longer time, mostly due to shared control system between the BCI and the simulated intelligent wheelchair [97].

Another SMR-based BCI was the one presented in [109], where 28 participants (14 control group patients and 14 suffering from Spinal Muscular Atrophy type II (SMA II) or Duchenne Muscular Dystrophy (DMD)) underwent BCI training in environment, targeting home area and had to control some domestic appliances such as neon lights and bulbs, TV and stereo sets, motorized bed, acoustic alarm, front door opener, telephone and wireless cameras (to monitor the different rooms of the house ambient).

One of the most important and the first BCI application enabling communication are spellers, which are meant for subjects unable to speak. Spellers can be P300-, SSVEP- or motor imagery-based (event-related (de)synchronization (ERD/ERS)) [96,110,111,112,113]. Despite being very basic, they are able to provide some kind of independence for patients, who are unable to communicate in any way, such those with the ALS or locked-in patients [96,112,114,115,116]. This is because most of the BCI paradigms rely on Event-Related Potentials (ERPs), such as among the others P300 and SSVEP [114,115,117,118].

The P300 paradigm is one of the most reliable techniques for BCI systems. They are also useful for more complex BCI applications [119]. The P300 can apply either standard (passive) or “oddball” paradigms (uncommon, unconventional stimulus) [120,121]. The use of P300 BCI requires very little user-training, which makes it easy to apply [18,112,116].

The P300 BCI appear in the following forms/implementations [112,116]:

- Classic P300 BCIs;
- P300 BCIs using tactile stimulation through small discs (tactors) places over specific areas;
- Hybrid P300-BCIs—combining various types of BCI systems;

One of the most popular P300 applications are the P300 spellers, which were first introduced in 1988 by Farwell and Donchin [69,116]. It is based on rare stimuli, which occur as a positive deflection 300 ms after it [116,117]. P300-based spellers require patient to choose appropriate character from a 6×6 or 5×5 matrix [112,114,116] or to control a wheelchair [117]. Another interesting P300 application is mouse emulation device (MED) for users with cervical spinal cord injury (SCI) with the accuracy of 82% and response time below 149 s. The study participants showed interest in using such BCI application on a regular basis [122]. The P300-BCI can also be applied in smart home applications, such as the one presented in [123]. Besides practical implementations of the P300-based Brain-Computer Interfaces, it is also important to mention systems designed typically for entertainment purposes—VR (virtual reality) gaming [124].

In [119] it has been shown that visual stimuli provide stronger P300 responses, which may enhance the difference between target and non-target responses, which may positively affect the accuracy and reliability of the P300-BCIs.

The current P300-based BCIs rely not only on P300 paradigms but also on other visual ERPs such as the N100, N200 or N400 components [115,119].

The SSVEP-based (steady state visual evoked potentials) Brain-Computer Interfaces are one of the most widely developed systems, mostly due to their non-invasiveness, high signal-to-noise-ratio and high-speed performance [118,125]. The SSVEP-BCIs also require little or not training, what makes it a great candidate for real-life applications [113,118]. The steady state visual evoked potentials (SSVEPs) are elicited by the same, repeated visual stimulus applied [18].

One of the interesting SSVEP-BCI applications are spellers, such as Shuffle Speller typing interface presented in [126], which could be also applied by ALS patients. In another study (see: [127] the authors adopted a QWERT style keyboard with flickering LEDs for the SSVEP-BCI. The data for the SSVEP-BCIs is usually measured from the occipital regions, however, interesting system using an in-ear electrode was presented in [125]. Such systems can also be used to control neuropstheses with the flickering lights mounted on it as presented in [128,129] (two-axes electrical hand proshesis control with the accuracy of 44–88%), or to control robotic arms or assistive robots [130].

The BCI systems enable also to control various external devices such as inter alia quad-copter. The solution presented in [131] applies inexpensive off-the-shelf Emotiv EPOC device for the purpose of real-time brain’s activity recording. Besides, the EEG signals, facial expression are also used in order to control the device.

Cho et al. presented in 2018 ([132]) an EEG-BCI system, which relied on decoding of five different real (ME—motor execution) and imagery (MI—motor imagery) hand movements. The experiment involved using common spatial patterns (CSP) and regularized linear discriminant analysis (RLDA). The data was analysed offline and the obtained results were as follows—56.83% for the ME, and 51.01% for the MI.

Despite the fact that the BCI systems are still not being widely used in everyday life, they provide a lot of important medical information for diagnostics purposes [18].

As it was presented above—the most popular Brain-Computer Interfaces are best on either EEG or intracortical recordings. This is mostly due to their portability and relatively low cost for implementation. Due to the lack of necessity of surgical intervention—the vast majority of the BCI systems are based on non-invasive scalp recorded signals such as the EEG [3,133,134]. Thus, it is important to mention also other measurement techniques such as among the others near-infrared spectroscopy or functional magnetic resonance imaging (fMRI), which are not very popular, but have their implementation in various applications—such as inter alia rehabilitation, and despite their higher cost are still being further investigated [82,83,134,135,136,137].

The fMRI-based BCI systems are one of the most important complements technology to the “family” of the non-invasive BCIs. Their main disadvantage is lack of portability, cost and challenging data analysis. Implementation of the fMRI is also uncomfortable for the patients, mostly due to the noise it generates [88,135]. However, it is the only method, which provides high spatial resolution data of the whole brain activity, where the EEG signals provide rather low spatial resolution [135,136,138].

The fMRI measures the blood oxygen level-dependent signals (BOLD) [136]. The fMRI technology is very advanced and allows volitional control of brain’s anatomically specific regions and it also can show some neurological disorders in these areas, such as inter alia [82]:

- chronic pain,
- motor diseases,
- psychopathy,
- social phobia,
- depression.

It is possible to classify the fMRI-based BCI systems into the four below listed categories [135]:

1. performance of higher-order cognitive tasks such as e.g., mental calculation,
2. language-related tasks conversion such as e.g., mental speech and/or mental singing,
3. performance of imagery tasks such as e.g., motor, visual, auditory, tactile, and emotion imagery,
4. performance of selective attention tasks such as e.g., visual, auditory, and tactile attention.

The fMRI-BCIs can be applied for the modifications in neurologically affected regions and treated in appropriate way, as unlike the EEG-BCIs—the fMRI-BCIs enable brain’s activity in very specific parts of its cortical and sub-cortical regions. The fMRI system is a typical closed-loop system [82].

As it was mentioned above, one of the main disadvantages of the fMRI-based BCI are its high cost, lack of portability and complexity of development and usage. Hopefully, with the rapid technological development such systems may become more popular in the near future [82,88,139,140,141].

Despite some disadvantages of the fMRI-based BCIs, they can be a good solution for patients with little or none functional recovery of upper limb motor function. It has also strong therapeutic potential for stroke rehabilitation, combined with more portable near infrared spectroscopy (fNIRS) [137,140,142]. Both BOLD and EEG data seem to be highly correlated, which is a good combination for hybrid EEG-fMRI-BCI systems [136].

Another interesting brain’s activity measurement method is functional near-infrared spectroscopy (fNIRS), which is a low-cost, non-invasive and portable technique [91,142,143,144]. Despite its lower spatial resolution to the one obtained from the fMRI and the lower temporal resolution to the one obtained from the EEG, it can be a good alternative to those two. It is because of its features such as among the others—low price and high portability and the fact that it can be used in nearly natural environments [143]. The fNIRS allows measurement of the oxyhemoglobin (HbO) and deoxyhemoglobin (HbR) concentration changes by picking two distinct near-infrared (NIR) wavelengths (600–1000 nm). It also offers subsecond temporal resolution and spatial resolution in 1 cm${}^{2}$. Its disadvantage is the response time for the commands execution compared to the EEG [91].

A good alternative in order to overcome the above mentioned issues are hybrid BCI systems combining various brain imaging methods, such as EEG-fNIRS BCIs [91,145,146] or EEG-fMRI [136,147].

**Figure 4:**
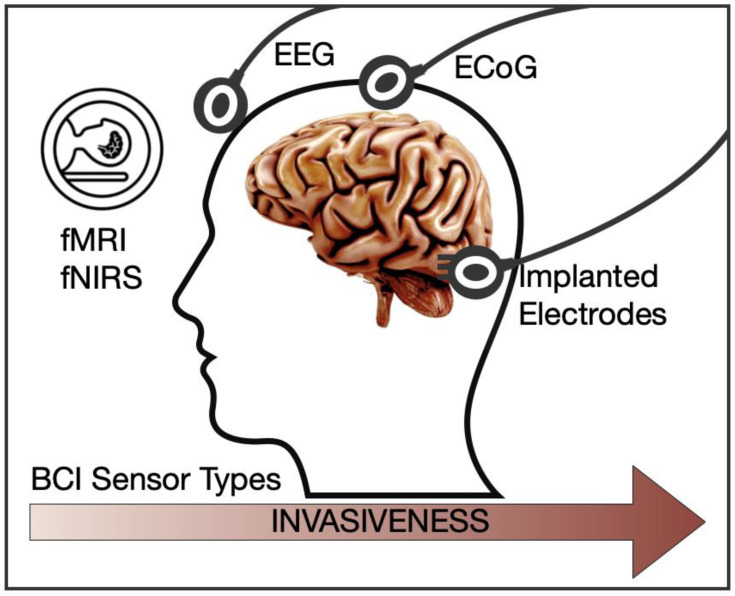
BCI-sensors types [148,149].

An overview of the most typical BCI sensors ordered by their invasiveness was illustrated with the Figure 4 and Table 2 [148,149].

### 2.4. BCI Systems—Recording Devices—Brief Review

Due to the inter alia high costs—commercial, non-invasive BCIs will remain limited to the public [96]. There is still drive in medicine or neurosciences towards smaller, portable, cost-effective and efficient devices [10].

The Brain-Computer Interface devices are becoming cheaper and more inconspicuous. The right choice of appropriate device is remarkably important for further research purposes. In 2018 Yu and Qi (see: [25]) conducted a consumer survey for choosing the best wearable non-invasive EEG-based BCI and the three top features for choosing appropriate headset were the following criteria:

1. safety—84.26%,
2. effect accuracy 59.34%,
3. wearing comfort 58.3%.

The most frequently applied headset are delivered by the following companies [25,150,151,152,153,154,155,156,157]:

- Emotiv Inc. (San Francisco, CA, USA),
- Ant Neuro (Hengelo, Netherlands),
- Cognionics (San Diego, CA, USA),
- Neurosky Inc. (San Jose, CA, USA),
- OpenBCI (Brooklyn, NY, USA),
- interaXon (Toronto, Canada),
- g.tec (Schiedlberg, Austria),
- CREmedical (Kingston, RI, USA).

**Figure 5:**
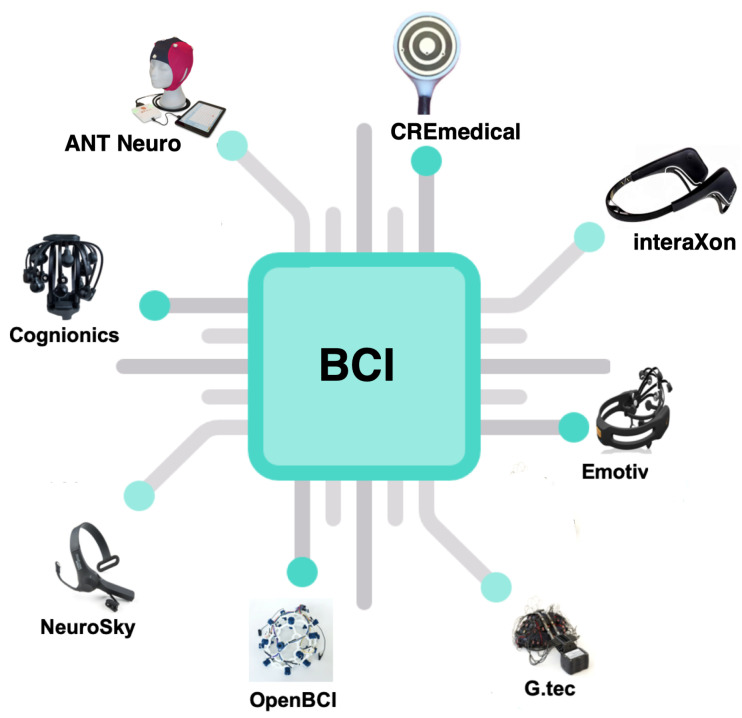
The most popular inexpensive, customer-grade EEG headsets from the Table 3 (based on [156,158]).

In Figure 5 the most popular off-the-shelf EEG headsets are illustrated. Their features list is outlined in Table 3 [156,158]. This is because one of the latest challenges in BCI development are both, software and hardware improvements in order to make them as user friendly as possible [159].

The Emotiv Inc. was founded in 2011 by tech entrepreneurs Tan Le and Dr. Geoff Mackellar, but they started their research already in 2003. The company is located in San Francisco—USA, with some branches located in Sydney (Australia), Hanoi (Vietnam), and Ho Chi Minh (Vietnam). Their products are intended to be used for research applications and personal use only. Emotiv is a well recognized pioneer and market leader in this field [25,150,151].

Applications for the Emotiv technology and interface span an amazing variety of potential industries and applications—from gaming to interactive television, everyday computer interactions, hands-free control system, smart adaptive environments, art, accessibility design, market research, psychology, learning, medicine, robotics, automotive, transport safety, defense, and security [25,150,151,163].

The Emotiv company released few devices [150,151,156,161,163]:

- Emotiv EPOC (2009) and Emotiv EPOC+ NeuroHeadset (2013)—14-channels device, with 2 referential sensors, wireless Bluetooth connection, battery, and a USB port;
- Emotiv Insight (2015)—a simpler 5-channel wireless EEG device, designed for everyday use with advanced electronics and full optimization, designed for everyday use by individuals;
- Emotiv EPOC Flex (2019)—equipped with 32 measuring sensors available in two options: gel- and saline-sensors. It has wireless technology, is elastic, and adjusts to the head shape;
- Emotiv EPOC X (2020)—14-channel wireless headset.

Their prices range from 299 USD to 1699 USD [161,163,164]. The Emotiv EPOC headset is very easy to use and does not require any particular scalp preparation [3,164]. Its disadvantage (for all versions) is that it does not provide much open source and has some applicability limitations, as it is very difficult to implement on it the SSEVP-BCI (Steady State Visual Evoked Potential) [164].

A very interesting study was the one where the Emotiv EPOC was compared with the clinically-approved device—g.tec. Comparison between the two devices proved the recorded data to be similar, although the g.tec provided stronger and cleaner signal [163,164].

Neurosky Inc. was founded in 2004, in Silicon Valley, California but they began to work on their technology development already in 1999. They deliver non-invasive, inexpensive EEG-based Brain-Computer Interfaces, where they also use EMG signals [25,150].

The Neurosky Inc. launched a couple of products, such as [25,150,156]:

- MindSet (2009),
- MindWave (2011),
- MindWave Mobile (2012),
- MindWave Mobile 2 (2018).

The biggest advantage of their products is a low, competitive price (starting from 99 USD) and ease of use. The Neurosky devices are also appropriate for mental monitoring of biometric data such as attention/meditation during sports or visual tasks [150,165].

In 2010—Crowley et al. carried out some psychological tests in order to induce stress and correlated the results with measured attention and meditation signals. The applied headset was Neurosky Mindset. The assessment was performed with the use of Stroop and Towers of Hanoi tests [25,150,152,160,166].

- electroencephalography (EEG),
- electromyography (EMG),
- electrocardiography (ECG).

Manufacturers have also released an open-source application for use with the device, which allows users to freely work with it [160]. The OpenBCI delivers 4 types of kits [150,160]:

1. 21-channel EEG Electrode Cap Kit (2019) with Ag/AgCl coated electrodes;
2. 16-channel All-in-One Biosensing R&D Bundle (2014) with different approaches EEG data acquisition:
  - dry electrodes—EEG Headset,
  - wet electrodes—gold cup electrodes;
3. 8-channel OpenBCI EEG Headband Kit (2018) with dry electrodes.
4. OpenBCI Galea (announced in Novemebr 2020)—combines mixed reality (XR) headsets with state-of-the-art biosensing and BCIs with several types of sensors:
  - electroencephalography (EEG),
  - electrooculography (EOG),
  - electromyography (EMG),
  - electrodermal activity (EDA),
  - photoplethysmography (PPG).

The prices vary from 199 USD to 2500 USD [25].

One of the interesting projects, where OpenBCI was applied was related with affective video selection and users’ emotions recognition. This was introduced by Lakhan et al. in 2019 in: [167].

Similar study was presented in [163]—the OpenBCI headset was compared with a clinically approved g.tec device g.USB. The medical grade equipment slightly outperformed the consumer grade one and the OpenBCI gave very close EEG readings to those obtained with the g.tec device [168]. The correlation between both temporal and spectral features showed that the signals acquired by both amplifiers were almost identical—very close. The obtained Pearson R score was greater than 0.99 [169].

InteraXon was founded in 2007 by Ariel Garten, Trevor Coleman, Chris Aimone, and Steve Mann [162]. The company is based in Toronto, Ontario, Canada. The aim of their product is to help patients reach a deep relaxation state. Their headsets are also widely used for various purposes such as health-related or scientific and medical research [25,162]. Their product’s price is about 250 USD [162]. They offered the following models [25,150,162]:

- Muse (2014)—a 7-sensors device designed with dry sensors, which do not require any liquid;
- Muse 2 (2018)—device with 4 EEG electrodes, heart sensors (PPG + Pulse Oximetry), accelerometer, and gyroscope.

Another BCI manufacturer is g.tec medical engineering created by Christoph Guger and Günter Edlinger in 1999 in Austria. The company develops and produces high-performance Brain-Computer Interface Systems and neurotechnologies for non-invasive and invasive recordings. The equipment provided by them is clinically approved and enables to register high quality data. Their current offer include the following products [155,164]:

- g.NAUTILUS PRO—available with prefixed 8/16/32 dry or wet EEG electrodes with 3-axis accelerometer.
- g.NAUTILUS RESEARCH—a hybrid (dry and wet EEG electrode) version and a gel EEG electrode version with 8/16/32/64 EEG channels. This device is non-certified (for potential clinical applications), which results in a lower price of this device for only neuroscience research.
- g.NAUTILUS fNIRS—it enables simultaneous recordings of both EEG and fNIRS (functional near-infrared spectroscopy) signals. It provides the top-quality EEG recordings from 64/32/16/8 g.SCARABEO EEG channels and 8 fNIRS channels within a few minutes.
- g.NAUTILUS MULTI PURPOSE—multiple EEG and biosignal amplifier, which can connect to other body sensors such as ECG/EOG/EMG electrodes to measure GSR, respiration, and many other biosignals.

One of the most popular clinically-approved, professional EEG systems from g.tec is g.USBAMP from the g.tec company. It is rather a pricey device (ca. 25 USD), which provides excellent data quality [155,164].

An alternative to traditional (dry and wet) EEG electrodes is a Tripolar EEG (tEEG), which is a new platform of the EEG device using innovative ultra-sensitive electrodes—the Tripolar Concentric Ring Electrodes (TCREs) in order to detect brain signals from the surface of the scalp [153,154,170].

The tEEG device was founded in 2017 by CREmedical corporation. Unlike conventional EEG, the tEEG can increase the resolution and the quality of the recorded brain signals, which is carried out while using their new electrodes (TCRE), which enables to perform artifacts suppression in real time [153,171,172]. Therefore, it can increase signal-to-noise ratio up to 375%, spatial selectivity up to 257%, and reduce overlap information with 8.3% [153,170,172].

Above—mostly inexpensive, off-the-shelf BCI systems were presented, which are popular mostly due to their price and availability, it is however, important to mention those applying clinical-grade equipment, mostly due to the high quality-signals they provide [3]. Based on authors’ professional experience and thorough literature studies, most of the clinical-quality EEG data for the BCI applications are gathered using the following clinical-grade amplifiers [18,107,155,173,174,175,176,177,178,179,180]:

- g.tec amplifiers;
- Porti7 (TMSI);
- Nuamp amplifier;
- BrainAmp128DC;
- BioNomadix amplifier (Biopac);

Clinical-level (medical devices) EEG equipment is also popular in numerous BCI-related applications. There is a wide range of cases where the g.tec ([155]) amplifiers are used, e.g., BCI systems dedicated for controlling a neuroprosthesis [173]. Another popular clinical-level device is Porti7 from the TMSI company, which was applied for a SSVEP-BCI system, where the authors tried to find the most appropriate SSVEP frequencies [174]. Similar study also with the use of Porti7 was presented in [176], where the authors tried to find the most appropriate frequency range of the SSVEPs and based on these EPs speller. Another study involving Porti7 device regarded comparison of various wet-electrodes (water- vs. gel-based) in SSVEP-based BCIs [175]. The neuroscan device—Nuamp was applied for a BCI-based post-stroke patients’ rehabilitation [177]. BrainAmp128DC was used in studies [178,180] for the data gathering for EEG-based robotic arm control. In [179] the authors compared inexpensive Neurosky’s Mindwave device with the Biopac’s BioNomadix amplifier, the obtained results proved similar quality of the recorded data (correlation factor between the power spectra of the both devices was greater than 0.7).

It is very hard to describe all medical devices, mostly due to the fact that in many cases they are very expensive and such a high quality specification may not be necessary for simple signal detection required for P300- and SSVEP-based BCIs. Numerous studies regarding data quality comparison between the inexpensive and professional equipment have been carried out and proved that those cost-efficient headset are able to provide signals of similar to clinical quality. In [163,168] the OpenBCI (inexpensive) was compared with a g.tec amplifier, and the study proved that the cos-efficient OpenBCI can record data of close to the professional quality. The obtained signals were identical and the Pearson R score was greater than 0.99. Similar comparison, but with different devices was presented in [179], where the Mindwave was compared with the BioNomadics. The power spectra of the obtained EEG signals were very similar. Such tests prove that rapid development of inexpensive-EEG-headset manufacturing results with high-quality, but cost-efficient devices.

## 3. The Newest Trends and Further Development Paths in BCIs

In the late 50s Bancaus and Talarach introduced sEEG (stereoencephalography) electrodes, which are invasive, deeply implanted EEG electrodes [181]. They were mostly applied for the purpose of epileptic zones detection [181,182,183,184,185]. They can be a great alternative to the popular ECoG-based systems, however, in some cases the both types of the measurement electrodes are combined [182]. The ECoG provides higher density data coverage, while the sEEG provides sparser coverage spanning more, bilateral brain regions including its deeper structures [182,186]. It is believed that the sEEG, despite not being the newest measurement technology, holds great potential for the BCI applications as it offers the measurement of brain structures, which cannot be reached with the ECoG, also, the sEEG enables to decode the memory-related processes and limbic activity, which can also be used in order to either supplement or further enhance decoding of other cognitive processes. The sEEG can be a future of invasive-BCIs [182,187].

One of the newest trends in the SSVEP-BCIS is application of combined spatial filtering algorithms. In [188] a framework consisting of these four elements: data, temporal filter, orthogonal projection and new spatial filter. The authors solution enables to study, explore, compare and improve spatial filtering algorithms in order to develop high performance SSVEP-based BCI systems.

The most efficient BCIs are claimed to be P300- and SSVEP-based. Unfortunatelly there is still no perfect, ideal Brain-Computer Interfsce, therefore, in order to overcome the limitations of many modern BCIs—the hybrid BCIs can be applied. The hybrid BCIs are systems, where traditional BCI is combined with another interface in order to have the system more versatile, usually it means combination of brain signals with other physiological data. One of the most promising solutions is a Hybrid BCI using Visual Evoked Potential (VEP)—called V-BCI. Some intiial studies showed that in the future the use of hybrid V-BCI in direct control mode in entertainment and quality of life applications will be highly demanded in particular for patients with neurological disorders [103].

The future directions of the BCI-related studies are strongly connected with the availability of the cost-efficient EEG headsets. The current tendency of sensor development focuses on signal quality improvement (by using more and more sophisticated signal processing methods) and also improvement of the comfort while using the system (material for sensors’ construction and design of various types of dry electrodes). The future directions also involve combination of the non-invasive BCIs with Augmented Reality (AR) systems, which are also becoming more and more available and cost-efficient [158]. The BCI technology itself is not the newest, however, its improvement and development is rapid [189].

Current and probably future trends in development of the BCI systems are strongly related with the development of intelligent algorithms for the analysis of biomedical data (in particular brain signals). There is also a need to improve the performance of the already used methods by calibration time reduction and classification accuracy improvement. The scientists are also trying to design and develop systems with reduced number of channels [159].

Another emerging trend in the development of the BCI systems are the passive BCIs. The traditional BCI systems are active or reactive, which means that the user has to be engaged in particular mental task, while the passive BCIs work more autonomously [190]. Based on this it is possible to divide the BCI systems into the following categories [189,191]:

1. Active BCIs—are controlled by the user through a specific mental task performance:
  - motor imagery—the user has to imagine movement of a limb, which can be later translated into appropriate command;
  - blinking—eye blinking registered in the EEG can be used as a control command.
2. Reactive BCIs—the user produces brain signals as a response to external stimulations such as visual or audio stimuli:
  - Event-Related Potential—natural brain’s response to a specified event or a stimulation;
  - Visual Evoked Potential—a form of ERP, which depends on visual stimuli.
3. Passive BCIs—a system, which focuses on the cognitive feedback of the users’ brains’ activity. The system works partially autonomous:
  - emotions—emotion recognition, recognized by the BCI system;
  - mental state—the BCI system is able to recognize and analyse the user’s mental state and provide him/her with appropriate feedback.

Table 4 presents brief summary of the most current trends, based on subjective authors’ choice [18,82,83,130,134,135,136,137,140,142,150,192,193,194,195,196,197,198,199,200,201,202,203,204,205,206,207,208,209,210,211,212,213,214,215,216,217,218,219,220,221,222,223,224,225]. It is impossible to list all trends, improvements or developments in this study area, as there are too many.

## 4. Advanced Signal Processing Methods for BCI Systems

The biological signals appear to be of random (stochastic) nature, so it is impossible to predict their value at any instant in time and therefore only some statistical measures can be used in order to determine some of their features. The stochastic signals can be divided into two groups [226,227]:

1. stationary:
  - ergodic,
  - non-ergodic.
2. non-stationary.

**Figure 6:**
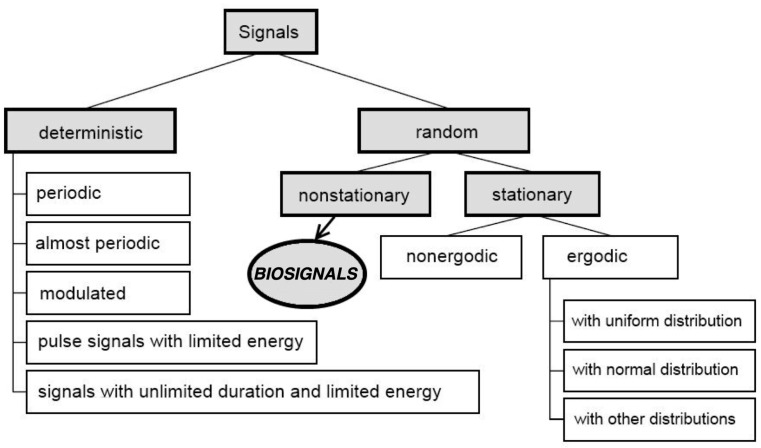
Basic scheme for classification of signals [226].

The brain signals used in the BCI system have typical biomedical data features, which allows to classify them as either, continuous or discrete [3,226,228]. In order to process these signals and classify them in the appropriate way it is important to conceptualise them and have awareness of what kind of data they actually are. In Figure 6 a very basic and simplified scheme of signals’ classification is shown [3,226].

The brain signals in their nature have spatio-temporal information. The BCIs are applied to process this information along with a specific task, which can be used e.g., for detection purposes. In fact, the performance of the BCI systems strongly depends on the quality of provided information (in this case—brain activity related signal) [146,229].

The electroencephalography (EEG) using classic disc electrodes can be qualified as a non-invasive technique used to record brain activities from the scalp and to discern temporal information but it leaves out spatial information [152]. Therefore a range of newer methods has been proposed in order to improve the overall quality of the brain signals, including e.g., those increasing signal-to-noise ratio (SNR) methods [3,13,153,154,230,231,232,233,234,235,236]:

1. conventional and high density EEG with different montages:
  - bipolar,
  - Laplacian,
  - common average references.
2. some methods of linear spatial filtering such as inter alia:
  - Principle Component Analysis (PCA),
  - Independent Component Analysis (ICA).
3. different hardware electrodes such as inter alia:
  - a bipolar electrode with five points finite difference method (FPM),
  - quasi-bipolar concentric electrode,
  - tri-polar concentric electrode.

The choice of appropriate electrodes types for the purpose of EEG data recording is of utmost importance, in particular when it comes to potential BCI implementation [152]. It is possible to distinguish two main categories of EEG electrodes [41,152,237,238]:

1. wet electrodes:
  - silver-chloride electrodes (Ag/AgCl):
    - low cost,
    - popular and widely used by current market products,
    - they have low contact impedance,
    - they require removing outer skin layer of the scalp and using conductor gels or pastes,
    - they require longer preparation time,
    - they may be uncomfortable for potential patients,
2. dry electrodes:
  - they do not require any type of skin preparation,
  - they do not need using any types of conductive gel or paste,
  - they may provide worse signal quality to the wet electrodes.

Biomedical data (in particular brain signals—EEG) are very challenging from the analytical viewpoint, mostly due to their non-stationary character and their low amplitude and low frequency range. Furthermore, these signals are often noisy and contaminated with various artifacts, which negatively affects their potential processing usability [3,13,230]. The EEG artifacts can be divided into the two following categories [3,13,152,239]:

- external:
  - Apparatus: broken electrode wire, bad contact of the electrode with the surface of the scalp, detachment of the electrode, etc.
  - power artifact: 50 Hz (Europe) or 60 Hz (US).
- internal—physiological artifacts generated by the body of the examined person:
  - EOG artifacts—caused by the eye movements;
  - cardiac artifacts—related to the ECG;
  - muscle artifacts—related to the EMG;
  - movement artifacts—caused by the subject’s body movements;
  - artifacts related to the sweat gland activity;
  - respiratory artifacts.

**Figure 7:**
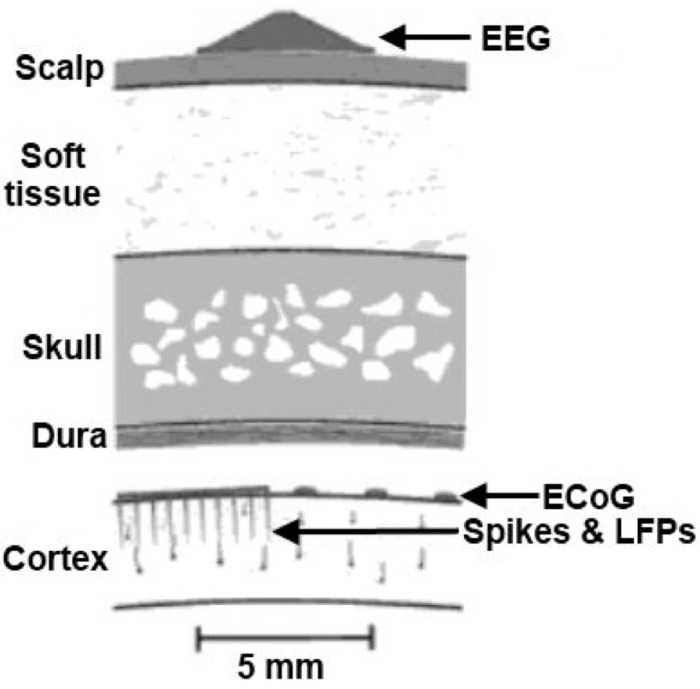
Layers the EEG signals have to go through [4].

Also, the overall quality of data is affected by the non-invasive way of their recording, as the signals have to “go” through multiple layers (see: Figure 7) [4].

The last few years have brought quite a significant development of advanced digital signal processing methods and also rapid development in the measuring devices comparment [12]. Analysis of biomedical data usually requires some kind of pre-processing, especially in order to obtain desired patterns. This is because these signals are of a very complex nature, they are non-stationary and can be contaminated with various disturbances. Unfortunately, despite numerous attempts, an ideal and versatile method for them is still non-existent [2,58,226].

A typical bio-signal can be expressed by the below simple Equation (1) [226,240,241,242]:

(1) x(t)=s(t)+n(t),

where:

x(t)—is the measured biosignal,

s(t)—the actual deterministic signal,

n(t)—the additive noise.

The main aim for the application for most of the signal processing methods is the removal of the noise (n(t)) from the analysed data [240,241]. Based on the thorough literature review and on the authors’ professional experience—the most popular methods applied for analysis of biomedical signals include the following [12,40,52,58,228,243,244]:

1. advanced/sophisticated signal processing methods:
  - discrete and continuous Fourier Transforms,
  - Wavelet Transforms (WT),
  - Time-Frequency Analysis (TFA),
  - Blind Source Separation (BSS) methods:
    - Principal Component Analysis (PCA),
    - Independent Component Analysis (ICA),
    - Empirical Mode Decomposition (EMD).
  - Fuzzy Logic.
  - Artificial Neural Networks:
    - Convolutional Neural Networks;
    - Deep Learning Networks.
2. basic/simple simple digital and adaptive filtering methods;
3. various modifications and combinations—the so-called “hybrid methods”.

Signal processing of brain signals requires implementation of various modern and advanced signal processing methods as they consist of the two components—signal and noise, where the signal is usually in the form of a waveform and the noise is the remaining part, which has to be eliminated in order to make the analysed data more legible [242].

The amount of noise in the signal is expressed by the signal-to-noise ratio (SNR), which is basically the ratio of the signal to noise, expressed in the dB units in accordance with the (2) [3,242,245]:

(2) $$SNR=20log\left(\frac{Signal}{Noise}\right)\left[dB\right].$$

The higher the SNR the better is the signal quality [242,245,246].

The most popular signal processing methods applied for the purpose of analysis of biomedical data are various Transforms, such as [3,26,40,53,58,227,240,246,247,248,249,250,251,252,253,254,255,256,257]:

- Fourier Transform (FT);
- Discrete Fourier Transform (DFT)—enables decomposition of discrete time signals into sinusoidal components, were their frequencies are multiples of a fundamental frequency;
- Fast Fourier Transform (FFT)—frequently applied in analysis of any deterministic bio-signal’s spectral content, which is also a faster version of the Fourier (FT) and the Discrete Fourier (DFT) Transform. It is not designed for short-duration signals;
- Short-Time Fourier Transform (STFT)—involves multiplication of the analysed signal by a short-duration time window, which is slid along the time axis of the signal in order to cover the whole duration of it and to obtain estimate of the signal’s spectral content. Within the short-duration window the signal is assumed to be stationary. The STFT can be also considered as a kind of method for signal filtering using a band-pass filter centered around a given frequency *f*, where the impulse response is the FT of the short-duration window modulated to that frequency. It is also known as Gabor Transform;
- Discrete Hartley Transform (DHT)—very popular in various BCI applications. It is similar to the DFT;
- Fast Hartley transform (FHT)—faster DHT, twice as fast as the FFT;
- the Discrete Cosine Transform;
- the Discrete Hilbert Transform;
- the Discrete Fractional Hilbert Transform;
- the Discrete-Time Wavelet Transform;
- the Discrete Walsh Transform;
- the Discrete Hadamard Transform;
- Wavelet Transforms (WT)—popular in processing of biomedical images and biomedical signals. Used for conversion of the complex signals from the time- into the frequency-domain. Is computationally heavy, which makes them unsuitable for implementation on embedded platforms. Contrary to the STFT the WT provides a more flexible way of signal’s time-frequency representation by allowing the use of variable sized windows. There are numerous types of Wavelet Transforms such as inter alia:
  - Continuous Wavelet Transform (CWT),
  - Discrete Wavelet Transform (DWT);
  - Tunable-Q Wavelet Transform.

The main aim of Fourier Transforms application is to transform the signals from the time-domain to the frequency-domain [3,248,258].

The main difference between the above mentioned Fourier Transforms and the Wavelet Transform is that the FT use windows of constant width and the WT use frequency-dependant windows [240]. The Wavelet Transforms enable arbitrarily good time resolution for the high-frequency components and arbitrarily good frequency resolution for the low-frequency components [240,243,255]. The Wavelet Transforms are particularly effective for non-stationary signals processing [26].

The analysis of biomedical data requires implementation of sophisticated signal processing methods such as wavelets, and in particular [3,26,40,58,212,240,243,255,259,260,261]:

- Morlet Wavelet—works well with signals with short duration of the high-frequency components and long duration of the low-frequency components, such as the EEG signal;
- Daubechies Wavelet function—were investigated for the analysis of epileptic EEG recordings;
- Harmonic Wavelet function—enables to achieve exact band separation in the frequency domain.

Wavelets can be defined as waves with limited duration and 0 average values. The Wavelets’ roots can be traced back to the thesis of Alfred Haar published in 1909, however, the broader concept of Wavelet was introduced by Alex Grossman and Jean Morlet in 1984 [26,58].

One of the most popular, well-known, but still efficient methods is the Time-Frequency Analysis (TFA), which can be applied not only for the purpose of analysis of biosignals, but also for other types of signals such as non-stationary, non-Gaussian signals, etc. [3,253]. The TFA relies on on cutting the signal into slice segments, which are later processed with e.g., Fourier Transform-based analysis, where in case of biosignals’ analysis—the segments would be interpreted as discontinuity [255,262]. The Time-Frequency analysis is a non-linear, quadratic transformation applied frequently for analysis of non-stationary signals. It uses both time and frequency functions [3,255].

The most popular windows applied in the TFA are [253,262]:

- Hamming,
- Hanning,
- Kaiser,
- Barlett.

Because every signal can be represented in form of any convenient set of orthogonal basis functions, which are its principal components [240,263]. The Principal Components Analysis (PCA) is another popular and advanced method applying mathematical principles to the signals, which transfer their correlated variables into principal components [264,265].

It has been developed before the World War II [264], but until today it is still an efficient method for removing various artifacts from biomedical signals [266,267,268]. It is also one of the simplest methods based on the BSS (Blind Source Separation) and its algorithm is based on the eigenvalues of the covariance matrix [243,263,269]. The PCA is sensitive to the original variables scaling [263].

The Principal Component Analysis (PCA) can be frequently implemented in analysis of biomedical signals such as EEG or ECoG [240,270]. One of the main disadvantages of the PCA is that using it with the extracted components is not always independent and invariant under transformation, which ends up with some classification assumptions and not real, desired results [271]. Sort of “improvement” of this method is the Independent Component Analysis (ICA), which is more flexible and which will be described in more detail in the next sub-subsection [243].

The Independent Component Analysis (ICA) is also a statistical method applied for the purpose of decomposition of a multi-variable signal into a set of mutually independent components [269]. The particular values of the signals are considered as samples of random variables and not as time functions [240,243,269].

As the ICA is kind of extended method of the PCA, however, it is more flexible and effective in artifacts removal from biosignals [240,243]:

- the source signals are statistically independent from each other and instantaneously mixed;
- the dimensions of the analysed signals have to be greater than or equal to the source signal;
- the sources

The ICA method, despite being so popular, has also some limitations [266,269]:

- only the original IC (Independent Component) can have the Gaussian distribution;
- only for the *n*-dimensional data vector it is possible to find a maximum of the *n*-dependent components with the use of the ICA method;
- it is impossible to determine the order of the original components with the ICA method.

The Empirical Mode Decomposition (EMD) is a method for pairs of signals decomposition, where one of them is introduced as a reference signal. It is a very suitable method for analysis of biomedical signals, which was introduced in 1998 [243,266]. The EMD method decomposes the signal s(n) into the sum of band-limited functions dm(n)—intrinsic mode functions (IMF) [243,266]. The EMD is an empirical and data driven technique, which means that, unlike other similar methods, it does not depend on basic functions selection such as inter alia the WT [243].

The EMD method is claimed to be one of the best methods for analysis of non-linear and non-stationary signals. It has however some limitations such as among the others the endpoint effect or modal aliasing, therefore it is frequently combined with other methods and applied as hybrid methods [272,273].

It is also possible do find BCI systems based on Fuzzy Logic (FL) [70,274,275,276,277,278,279]. The Fuzzy Logic provides more flexibility in the decisions making process as it has many facets, which can be inter alia as follows [280]:

- logical,
- fuzzy-set-theoretic,
- relational,
- epistemic.

It is very rare that the raw EEG data is possible to be analysed, mostly due to the presence of various contamination, artifacts and disturbances [3,13,230]. Appropriate filtering applications reduces noise, unwanted signal components and improves the SNR [13,226]. It is possible to distinguish the four main types of filters [3,242]:

1. low-pass filters—exclude the unwanted higher values in the signal;
2. high-pass filters—exclude the unwanted lower values in signals;
3. band-pass filters—pass signals within a certain range of frequencies without distorting the input signal or introducing extra noise;
4. band-stop filters (notch)—reject signals within a specific frequency band called the stop band frequency range and passes the signals above and below this band.

The most popular classical filters are the following [3,226,281]:

1. Butterworth,
2. Chebyshev (Type and II),
3. Elliptic,
4. Bessel.

Along with the four above mentioned filters it is possible to use one of four kinds of approximation—required by the characteristic’s modulus. On the choice of approximation depends the presence or lack of ripple in the filtering band, which is unfortunately impossible to be avoided [226,281].

As it was mentioned above—it is very hard to find perfect filters without a riddled characteristic. Also the integer order limits flexibility of the filter design, what can be solved with the use of non-integer order (fractional) filters. It his also frequently applied for the purpose of biomedical signals’ analysis, mostly due to enabling flexibility in filter shaping. Such filters are well grounded, however, their implementation still has some disadvantages [282].

The application of such filters is becoming more and more popular. The theoretical background of these filters is very well-documented and they were developed long ago—being first introduced in the 19th century [283,284], despite becoming popular recently [285].

The non-integer order systems enable detection of the waves in robust biomedical signals, but they also enable to model them [282,283,284].

The fractional order calculus means the calculation of non-integer order derivatives, which is an extension and kind of improvement of an ordinary differential calculus [286]. The non-integer based systems are mainly applied for control purposes [285,287], however, they are also applied for modeling of biological systems, in HIV therapy or in order to predict the dynamics of the hepatitis C virus spreading [283,286].

Another type of useful filtering method applied on the EEG data are smoothing filters as they do not affect the data in a negative way, which means important information removal. The smoothing filters make also the biomedical data more legible for medical professionals [13].

The most popular smoothing filters are the following [13,286,288,289]:

- Savitzky-Golay filter (S-G),
- Median filter,
- Bessel smoothing filter.

The Savitzky-Golay filter is one of the most popular and efficient smoothing filters, it is a simplified method for differentiation calculations, which smooths the data based on a least-squares technique [13,288]. It is a generalized moving average digital polynomial filter, which works in a way that each value is replaced with a new value previously obtained form a polynomial fitting, which is performed with a basic linear least-square fitting to the 2n+1 neighboring points, where the value *n* should be equal or greater than the order of the above mentioned polynomial. The more neighbors are applied the smoother the final signal is [288].

The Savitzky-Golay filters are usually applied for both, differentiation and smoothing. Their properties have also been very deeply studied and have been popular for over 50 years [290,291,292].

The median filter is on the other hand a non-linear filter, in which the mean value of a sequence of the processed point and its surroundings is measured. The advantage of this filter is that all of the values that deviate from the average are omitted and the output signal consists of the individual median values of all windows. The filter can be applied on both offline and online data using the moving median algorithm, which is similar to the moving average [13,293].

Both median and SG filters provide good results in filtering of various types of the data [13,196,292,293].

It is good to mention also some spatial filters (in particular the Laplacian filters), which are frequently applied for the purpose of the EEG data analyses [294].

Some of the authors of this work decided to develop an alternative to filtering of EEG signal and designed innovative threshold-base method, which does not involve filtering and does not affect the EEG data in a negative way [3,230].

In order to have a broader view on the most popular EEG signals’ processing methods it is important to mention the Artificial Neural Networks (ANN), which have been widely applied for the purposes of ECG and EEG classification of over twenty years [40,81]. One of the first attempts in using ANN in analysis of EEG signals was performed in 1994 by Tsoi et al. [52,295].

Implementation of traditional neural networks (NN) has been a part of scientific interests of many researchers for many years. The most recent has become developments of Deep Learning (DL) for such purposes, especially in case of large data-sets analysis, where the traditional NN had some difficulties, which resulted in development of the DNNs (Deep Neural Networks) [87,296,297].

The most popular architectures of the DNNs are the following [87,296,297,298,299,300,301,302]:

- Convolutional Neural Network (CNN)—relies on linear operation known as convolution. Provides good results during processing of images, audio, video and biomedical signals such as EEG;
- Recurrent Neural Network (RNN)—This type of network involves inbuilt memory cells for preserving the previous output states and uses it for processing purposes.

The implementation of the RNN for BCI systems decoding seems to be a perfect solution as it has network dynamics, computation and is non-linear and distributed. Unfortunately their BCI-applicability is limited, mostly due to their complexity in training [300]. In order to use their positive features, some modifications have been proposed such as inter alia multiplicative recurrent neural network (MRNN), as they are recurrent, but easier to train and has been found to be a good method for neuroprotheses [301].

In [302] the DNN model decoder was applied for controlling functional electrical stimulation (FES) of the participants’ paralyzed forearm. The implemented DNN model was at first trained offline using the concatenated imagined six-movement dataset. The obtained results were promising and showed good work of these system offline, however, due to such networks’ complexity, some further investigations regarding their online efficiency have to be carried out.

## 5. Discussion and Conclusions

This paper is an attempt to summarise over half century of Brain-Computer Interfaces—mostly because of the electroencephalography influence on these systems [2,54]. Over the time, numerous Brain-Computer Interfaces (invasive and non-invasive) have been developed, described and tested. Non-invasive nature of the EEG-based BCIs made them the most popular BCI systems [54,303]. Their application potential is vast and ranges from clinical to home-entertainment applications [10,303].

Their main purpose was to enable direct, non-muscular communication for handicapped people and later came solutions destined for gaming (pure entertainment), were followed by issuing various inexpensive, consumer-grade headsets [3,10,160,161,230,303,304].

To sum it all up—the BCIs can be applied to education and training, entertainment and neurogaming, medical assistance (e.g., spelling program, a motorized wheelchair, or neuroprostheses or exoskeletons), and/or emotional testing after appropriate development [11,23,25,214]. Also, the recent times show increased focus on the real-world applications of the BCI technology which speed up the transition of the BCI research from the laboratory to clinical products useful in everyday life.

Potentially, the BCI users might be individuals who are severely disabled by disorders such as inter alia [3,55,304,305,306]:

- ALS (Amyotrophic Lateral Sclerosis),
- cerebral palsy,
- brainstem stroke,
- spinal-cord injuries,
- muscular dystrophies,
- chronic peripheral neuropathies,
- psychiatric disorders.

Meanwhile, it is still important to develop and solve problems in the three critical areas [23]:

1. signal-acquisition hardware,
2. BCI validation and dissemination,
3. reliability.

As the EEG signals are prone to occurrence of various artifacts and disturbances it is difficult to analyse them and extract relevant information from such sensitive data [3]. Also, credibility of the obtained results and of the proposed solutions are not very straightforward. In many cases they appeared to be disappointing for people who gave hope to them [2].

The whole scientific area related to the BCIs is challenging, ungrateful and hard to follow. Also, after the rapid development in the 1990s, it is now difficult to find some spectacular breakthroughs. Despite some difficulties the Brain-Computer Interface-related research and development is source of tremendous excitement for scientists, engineers, clinicians, and individuals in general [23]. The market offers a solution for each of them, it is possible to fit an appropriate device for a particular user’s needs. Evidently, the recently implemented wireless, lightweight, and easy-to-use wearability has fashioned an impact on the ascending attractiveness of the non-invasive consumer-grade EEG devices among researchers from various fields of study [307,308].

One of the main disadvantages of this work is that it does not fully cover all new findings and all applied methods in the field, but it would be simply impossible—mainly due to the rapid development and growing interest in this scientific domain, so the authors were enforced to make something difficult choices regarding whether or not to include some of the most important systems/solutions, based on their subjective decisions.

To sum it all up, again, the BCIs took fiction, known from the sci-fiction literature, into reality by providing some ways to use “thoughts” for the control purposes [189]. However, although the systems are becoming more and more achievable, there is still a problem which makes it difficult to bring them out of the laboratories into daily life. And the problem is the convenience factor: long calibration time, using abrasive paste or gel to improve conduction and time related to placing the headsets on scalp are on the verge or slightly beyond the average user acceptance. Ultimately, some of these issues can be difficult to deal with by healthy users and almost impossible to deal with by people with various motor impairments [96,309]. When these obstacles will have been resolved, the BCIs will become a real part of our lives [309].

From the philosophical and ethical point of view concerning the brain-machine interactions raise many questions regarding distribution and attribution of responsibility, decision making and this is also designing a step toward construction artificial intelligence [310]. The interaction between brain and computer can help to explain intentions—the important attribute feature of human goal-directed behavior and have been debated commonly in the philosophy of mind [311]. 

## Figures and Tables

**Table 1:** Frequency ranges of the EEG signal [26,56,57,58].

Brainwave	Frequency Range	Mental Condition
Delta	0–4 Hz	State of deep sleep, when there is no focus, the person is totally absent, unconscious.
Theta	4–8 Hz	Deep relaxation, internal focus, meditation, intuition access to unconscious material such as imaging, fantasy, dreaming.
Low Alpha	8–10 Hz	Wakeful relaxation, consciousness, awareness without attention or concentration, good mood, calmness.
High Alpha	10–12 Hz	Increased self-awareness and focus, learning of new information.
Low Beta	12–18 Hz	Active thinking, active attention, focus towards problem solving, judgment and decision making.
High Beta	18–30 Hz	Engagement in mental activity, also alertness and agitation.
Low Gamma	30–50 Hz	Cognitive processing, senses, intelligence, compassion, self-control.
High Gamma	50–70 Hz	Cognitive tasks: memory, hearing, reading and speaking.

**Table 2:** Summary of the most popular sensors applied in BCIs [148].

Sensor	Type	Spatial Resolution	Temporal Resolution	Portability
micro-electrode	YES	0.05–0.50 mm	3 ms	moderate
ECoG	YES	1.0 mm	5 ms	good
intravascular electrode	YES	2.4 mm	5 ms	good
EEG	NO	2.4 mm	50 ms	good
fMRI	NO	>1 mm	1 s	poor
fNIRS	NO	1 cm	1 s	good
MEG	NO	>1 mm	1–5 ms	poor
PET	NO	3–51 mm	50–100 s	poor

**Table 3:** Some of the most popular inexpensive, off-the-shelf BCI systems—summary table [154,156,157,158,160,161,162].

Manufacturer	Wearable	Sensors Type	Channels Amount	Sampling Rate	Data Transfer
Neurosky	YES	Dry	1	500 Hz	Bluetooth
Emotiv	YES	Wet/Dry	5–32	500 Hz	Bluetooth
OpenBCI	YES	Wet/Dry	8–21	250–500 Hz	Bluetooth
ANT Neuro	YES	Dry	32–256	<16 kHz	Wi-Fi
g.tec	YES	Wet/Dry	8–256	500 Hz	Cable/Wi-Fi
Cognionics	YES	Dry	8–128	>2 kHz	Bluetooth
CREmedical	YES	Wet	20	500 Hz	Cable
interaXon	YES	Wet	4–7	250 Hz	Bluetooth
Cognionics	YES	Wet	8–128	<2 kHz	Bluetooth

**Table 4:** The summary of the most popular BCI applications.

Application	Description	Source Data	Analysis Method
spelling applications	One of the most basic applications of BCIs for people with disabilities used for communication purposes, where users using their brain activity choose appropriate letter [192,193,194,195].	EEG, EOG	P300 evoked potentials
neurogaming and VR/AR	Controlling video games, virtual and/or augmented reality applications using BCIs. It is one of the most popular current trends in the field [196,197,198,199,200].	EEG, EOG, EMG, heart-rate, motion control, facial expression	mVEPs, AI, DNN
neuromarketing	Neuromarketing methods, including EEG analysis, provide a better understanding of brain mechanisms and consumer behavior to improve marketing strategies [201,202,203,204,205].	EEG, EOG, facial expression, heart-rate	AI, DNN, various pattern recognition-methods
smart wheelchairs	One of the most needed BCI applications is the ability to control a wheelchair. Such devices are destined for people with cognitive/motor/sensory impairments [206,207,208,209,210,211].	EEG, EOG, heart-rate, facial expressions	DL, P300, SSVEP, EMD
emotional condition	Recognition of human emotions and/or mental states using biomedical data analysis—part of passive BCIs. Most of them are based on facial expression recognition and analysis of speech signals. It is one of the future development paths of BCIs [150,212,213,214,215,216].	EEG, heart-rate, EOG, EMG, facial expression, speech	DWT, AI, DNN, various pattern recognition methods (e.g., KNN, LDA), SSVEPs, Fuzzy Systems
robotics	Improvement of multidimensional control systems with the use of BCIs [130,217,218,219].	EEG, EMG, EOG	AI, DNN, CNN
’smart’ appliances	Controlling of various domestic appliances using BCIs, such as among the others window shutters, lighting, ambiance music, TV set screens, etc. or for connecting reality with AR solution [200,220,221,222,223,224].	EEG, EOG, heart-rate, speech	SSVEPs, P300, Fuzzy Systems, AI
rehabilitation	Good solution for patients with little or none functional recovery of upper limb motor function. It has strong therapeutic potential for inter alia stroke patients [18,82,83,134,135,136,137,140,142,225].	EEG, EOG, ECoG, fMRI, fNIRS, speech	SSVEPs, P300, EP, Fuzzy Systems, AI

## Data Availability

N/A.

## Abbreviations

The following abbreviations are used in this manuscript (in alphabetical order):

ADD	Attention Deficit Disorder
ADHD	Attention-Deficit/Hyperactivity Disorder
ALS	Amyotrophic Lateral Sclerosis
ANN	Artificial Neural Networks
AR	Augmented Reality
ASSR	auditory steady-state response
BCI	Brain-Computer Interfaces
BCS	Brain-inspired Cognitive System
BOLD	blood oxygen level-dependent signals
BSS	Blind Source Separation
CNN	Convolutional Neural Network
CNS	central nervous system
CWT	Continuous Wavelet Transform
DC	direct current
DFT	Discrete Fourier Transform
DHT	Discrete Hartley Transform
DL	Deep Learning
DNN	Deep Neural Networks
DSP	digital signal processing
DBD	Duchenne Muscular Dystrophy
DWT	Discrete Wavelet Transform
ECG	Electrocardiography
EDA	electrodermal activity
ECoG	electrocorticography
EEG	electroencephalography
EMD	Empirical Mode Decomposition
EMG	electromygraphy
EOG	electrooculography
ERD	event related desynchronisation
ERP	event-related potentials
ERS	Event-Related Synchronisation
FES	functional electrical stimulation
FFT	Fast Fourier Transform
FHT	Fast Hartley transform
FL	Fuzzy Logic
fMRI	functional resonance imaging
fNIRS	functional infrared spectroscopy
FT	Fourier Transform
HMI	Human-Machine Interfaces
ICA	Independent Component Analysis
LIS	Locked-in Syndrome
MEG	magnetoencephalography
ME	motor execution
MI	motor imagery
mVEPs	motion-onset visually evoked potentials
NN	Neural Networks
PCA	Principal Component Analysis
PD	Parkinson’s Disease
PET	positron emission tomography
PPG	hotoplethysmography
RNN	Recurrent Neural Network
SCP	slow cortical potentials
sEEG	stereoencephalography
SG	Savitzky-Golay filter
SMR	sensorimotor rhythm
SNR	signal-to-noise ratio
SMA II	Spinal Muscular Atrophy type II
SSVEP	steady-state visual evoked potentials
STFT	Short-Time Fourier Transform
TFA	Time-Frequency Analysis
VR	virtual reality
XR	mixed reality
WT	Wavelet Transform

## References

[1] Shortliffe E.H., Barnett G.O. (2006). Biomedical data: Their acquisition, storage, and use. Biomedical Informatics.

[2] Kübler A. (2019). The history of BCI: From a vision for the future to real support for personhood in people with locked-in syndrome. Neuroethics.

[3] Kawala-Janik A. (2013). Efficiency Evaluation of External Environments Control Using Bio-Signals. Ph.D. Thesis.

[4] Wolpaw J., Wolpaw E.W. (2012). Brain-Computer Interfaces: Principles and Practice.

[5] Ma T., Li H., Deng L., Yang H., Lv X., Li P., Li F., Zhang R., Liu T., Yao D. (2017). The hybrid BCI system for movement control by combining motor imagery and moving onset visual evoked potential. J. Neural Eng..

[6] Van Dokkum L., Ward T., Laffont I. (2015). Brain computer interfaces for neurorehabilitation–its current status as a rehabilitation strategy post-stroke. Ann. Phys. Rehabil. Med..

[7] Castro N., Siew C.S. (2020). Contributions of Modern Network Science to the Cognitive Sciences: Revisiting research spirals of representation and process. Proc. R. Soc. A.

[8] Wang Y., Kwong S., Leung H., Lu J., Smith M.H., Trajkovic L., Tunstel E., Plataniotis K.N., Yen G.G., Kinsner W. (2020). Brain-Inspired Systems: A Transdisciplinary Exploration on Cognitive Cybernetics, Humanity, and Systems Science Toward Autonomous Artificial Intelligence. IEEE Syst. Man Cybern. Mag..

[9] Schirmann F. (2014). “The wondrous eyes of a new technology”—A history of the early electroencephalography (EEG) of psychopathy, delinquency, and immorality. Front. Hum. Neurosci..

[10] Martins N.R., Angelica A., Chakravarthy K., Svidinenko Y., Boehm F.J., Opris I., Lebedev M.A., Swan M., Garan S.A., Rosenfeld J.V. (2019). Human brain/cloud interface. Front. Neurosci..

[11] Collinger J.L., Wodlinger B., Downey J.E., Wang W., Tyler-Kabara E.C., Weber D.J., McMorland A.J., Velliste M., Boninger M.L., Schwartz A.B. (2013). High-performance neuroprosthetic control by an individual with tetraplegia. Lancet.

[12] Theis F.J., Meyer-Bäse A. (2010). Biomedical Signal Analysis: Contemporary Methods and Applications.

[13] Kawala-Sterniuk A., Podpora M., Pelc M., Blaszczyszyn M., Gorzelanczyk E.J., Martinek R., Ozana S. (2020). Comparison of smoothing filters in analysis of EEG data for the medical diagnostics purposes. Sensors.

[14] Milanizadeh S., Safaie J. (2020). EOG Based HCI System for Quadcopter Navigation. IEEE Trans. Instrum. Meas..

[15] Saravanakumar D., Reddy R. (2020). A high performance asynchronous EOG speller system. Biomed. Signal Process. Control.

[16] Li K., Zhang J., Wang L., Zhang M., Li J., Bao S. (2020). A review of the key technologies for sEMG-based human-robot interaction systems. Biomed. Signal Process. Control.

[17] Yao D., Qin Y., Hu S., Dong L., Vega M.L.B., Sosa P.A.V. (2019). Which reference should we use for EEG and ERP practice?. Brain Topogr..

[18] Bamdad M., Zarshenas H., Auais M.A. (2015). Application of BCI systems in neurorehabilitation: A scoping review. Disabil. Rehabil. Assist. Technol..

[19] Epstein R. (2016). The empty brain. Aeon.

[20] Hassan M.A., Rizvi Q.M. (2019). Computer vs human brain: An analytical approach and overview. Computer.

[21] Collinger J.L., Kryger M.A., Barbara R., Betler T., Bowsher K., Brown E.H., Clanton S.T., Degenhart A.D., Foldes S.T., Gaunt R.A. (2014). Collaborative approach in the development of High-Performance Brain–Computer interfaces for a neuroprosthetic arm: Translation from animal models to human control. Clin. Transl. Sci..

[22] Miller K.J., Hermes D., Staff N.P. (2020). The current state of electrocorticography-based brain–computer interfaces. Neurosurg. Focus.

[23] Shih J.J., Krusienski D.J., Wolpaw J.R. (2012). Brain-computer interfaces in medicine. Mayo Clinic Proceedings.

[24] Zhang H., Wang H. (2008). Study on classification and recognition of multi-lead EEG signals. Comput. Eng. Appl..

[25] Yu X., Qi W. A user study of wearable EEG headset products for emotion analysis. Proceedings of the 2018 International Conference on Algorithms, Computing and Artificial Intelligence.

[26] Adeli H., Zhou Z., Dadmehr N. (2003). Analysis of EEG records in an epileptic patient using wavelet transform. J. Neurosci. Methods.

[27] Leuthardt E.C., Miller K.J., Schalk G., Rao R.P., Ojemann J.G. (2006). Electrocorticography-based brain computer interface-the Seattle experience. IEEE Trans. Neural Syst. Rehabil. Eng..

[28] Dubey A., Ray S. (2019). Cortical Electrocorticogram (ECoG) is a local signal. J. Neurosci..

[29] Wang P.T., King C.E., McCrimmon C.M., Lin J.J., Sazgar M., Hsu F.P., Shaw S.J., Millet D.E., Chui L.A., Liu C.Y. (2016). Comparison of decoding resolution of standard and high-density electrocorticogram electrodes. J. Neural Eng..

[30] Chakrabarti S., Sandberg H.M., Brumberg J.S., Krusienski D.J. (2015). Progress in speech decoding from the electrocorticogram. Biomed. Eng. Lett..

[31] Graimann B., Allison B.Z., Pfurtscheller G. (2010). Brain-Computer Interfaces: Revolutionizing Human-Computer Interaction.

[32] Villamar M.F., Al-Bakri A.F., Haddix C., Albuja A.C., Bensalem-Owen M., Sunderam S. (2018). T157. Seizure prediction with autonomic measurements versus intracranial EEG in patients with refractory epilepsy. Clin. Neurophysiol..

[33] Wittevrongel B., Khachatryan E., Carrette E., Boon P., Meurs A., Van Roost D., Van Hulle M.M. (2020). High-gamma oscillations precede visual steady-state responses: A human electrocorticography study. Hum. Brain Mapp..

[34] Amaral P., Paulo J., Cunha S., Dias P., Maria J. (2007). Multimodal Application for Visualization and Manipulation of Electrocorticography Data.

[35] Kingwell K. (2012). Neurally controlled robotic arm enables tetraplegic patient to drink coffee of her own volition. Nat. Rev. Neurol..

[36] Ethier C., Oby E.R., Bauman M.J., Miller L.E. (2012). Restoration of grasp following paralysis through brain-controlled stimulation of muscles. Nature.

[37] Millett D. (2001). Hans Berger: From psychic energy to the EEG. Perspect. Biol. Med..

[38] Gloor P. (1969). Hans Berger on electroencephalography. Am. J. EEG Technol..

[39] Berger H. (1938). Über das Elektrenkephalogramm des Menschen. XIV. Archiv für Psychiatrie und Nervenkrankheiten.

[40] Ocak H. (2009). Automatic detection of epileptic seizures in EEG using discrete wavelet transform and approximate entropy. Expert Syst. Appl..

[41] Lopez-Gordo M.A., Sanchez-Morillo D., Valle F.P. (2014). Dry EEG electrodes. Sensors.

[42] Beatty J., Greenberg A., Deibler W.P., O’Hanlon J.F. (1974). Operant control of occipital theta rhythm affects performance in a radar monitoring task. Science.

[43] Tudor M., Tudor L., Tudor K.I. (2005). Hans Berger (1873–1941)–the history of electroencephalography. Acta Medica Croat. Cas. Hravatske Akad. Med. Znan..

[44] Haas L.F. (2003). Hans berger (1873–1941), richard caton (1842–1926), and electroencephalography. J. Neurol. Neurosurg. Psychiatry.

[45] Coenen A., Zayachkivska O. (2013). Adolf Beck: A pioneer in electroencephalography in between Richard Caton and Hans Berger. Adv. Cogn. Psychol..

[46] Kułak W., Sobaniec W. (2006). Historia odkrycia EEG. Neurol. Dziecięca.

[47] Marshall L.H., Magoun H.W. (2013). Discoveries in the Human Brain: Neuroscience Prehistory, Brain Structure, and Function.

[48] Babkin B. (1946). Sechenov and Pavlov. Russ. Rev..

[49] Grigoriev A., Grigorian N. (2007). IM Sechenov: The patriarch of Russian physiology. J. Hist. Neurosci..

[50] Stone J.L., Hughes J.R. (2013). Early history of electroencephalography and establishment of the American Clinical Neurophysiology Society. J. Clin. Neurophysiol..

[51] Ebersole J.S., Pedley T.A. (2003). Current Practice of Clinical Electroencephalography.

[52] Hazarika N., Chen J.Z., Tsoi A.C., Sergejew A. (1997). Classification of EEG signals using the wavelet transform. Signal Process..

[53] Aydemir E., Tuncer T., Dogan S. (2020). A Tunable-Q wavelet transform and quadruple symmetric pattern based EEG signal classification method. Med. Hypotheses.

[54] Shahriari Y., Besio W., Hosni S.I., Zisk A.H., Borgheai S.B., Deligani R.J., McLinden J. (2020). Electroencephalography. Neural Interface Engineering.

[55] Wojcik G.M., Masiak J., Kawiak A., Schneider P., Kwasniewicz L., Polak N., Gajos-Balinska A. (2018). New protocol for quantitative analysis of brain cortex electroencephalographic activity in patients with psychiatric disorders. Front. Neuroinform..

[56] Ursuţiu D., Samoilă C., Drăgulin S., Constantin F.A. (2018). Investigation of music and colours influences on the levels of emotion and concentration. Online Engineering & Internet of Things.

[57] Robin M. (2009). A Handbook for Yogasana Teachers: The Incorporation of Neuroscience, Physiology, and Anatomy Into the Practice.

[58] Akin M. (2002). Comparison of wavelet transform and FFT methods in the analysis of EEG signals. J. Med. Syst..

[59] Jurcak V., Tsuzuki D., Dan I. (2007). 10/20, 10/10, and 10/5 systems revisited: Their validity as relative head-surface-based positioning systems. Neuroimage.

[60] Rangayyan R.M. (2015). Biomedical Signal Analysis.

[61] Merletti R., Parker P.J. (2004). Electromyography: Physiology, Engineering, and Non-Invasive Applications.

[62] Fajkus M., Nedoma J., Martinek R., Vasinek V., Nazeran H., Siska P. (2017). A non-invasive multichannel hybrid fiber-optic sensor system for vital sign monitoring. Sensors.

[63] Sidikova M., Martinek R., Kawala-Sterniuk A., Ladrova M., Jaros R., Danys L., Simonik P. (2020). Vital Sign Monitoring in Car Seats Based on Electrocardiography, Ballistocardiography and Seismocardiography: A Review. Sensors.

[64] Clerc M., Bougrain L., Lotte F. (2016). Brain-Computer Interfaces.

[65] Weisz N., Schandry R., Jacobs A.M., Mialet J.P., Duschek S. (2002). Early contingent negative variation of the EEG and attentional flexibility are reduced in hypotension. Int. J. Psychophysiol..

[66] Walter W.G., Cooper R., Aldridge V., McCallum W., Winter A. (1964). Contingent negative variation: An electric sign of sensori-motor association and expectancy in the human brain. Nature.

[67] Sterman M.B., Howe R.C., Macdonald L.R. (1970). Facilitation of spindle-burst sleep by conditioning of electroencephalographic activity while awake. Science.

[68] Kuhlman W.N. (1978). Functional topography of the human mu rhythm. Electroencephalogr. Clin. Neurophysiol..

[69] Farwell L.A., Donchin E. (1988). Talking off the top of your head: Toward a mental prosthesis utilizing event-related brain potentials. Electroencephalogr. Clin. Neurophysiol..

[70] Nguyen T., Hettiarachchi I., Khosravi A., Salaken S.M., Bhatti A., Nahavandi S. Multiclass EEG data classification using fuzzy systems. Proceedings of the 2017 IEEE International Conference on Fuzzy Systems (FUZZ-IEEE).

[71] Arafat I. (2013). Brain-Computer Interface: Past, Present & Future.

[72] Kolhe S., Khemani D., Bhatt C., Dubey N. (2018). Automation of appliances using electro-encephalography. Emerging Technologies for Health and Medicine: Virtual Reality, Augmented Reality, Artificial Intelligence, Internet of Things, Robotics, Industry 4.0.

[73] Birbaumer N., Ghanayim N., Hinterberger T., Iversen I., Kotchoubey B., Kübler A., Perelmouter J., Taub E., Flor H. (1999). A spelling device for the paralysed. Nature.

[74] Rezeika A., Benda M., Stawicki P., Gembler F., Saboor A., Volosyak I. (2018). Brain–computer interface spellers: A review. Brain Sci..

[75] Hochberg L.R., Bacher D., Jarosiewicz B., Masse N.Y., Simeral J.D., Vogel J., Haddadin S., Liu J., Cash S.S., Van Der Smagt P. (2012). Reach and grasp by people with tetraplegia using a neurally controlled robotic arm. Nature.

[76] Gollahalli A.R. (2015). Brain-Computer Interfaces for Virtual Quadcopters Based on a Spiking-Neural Network Architecture-Neucube. Ph.D. Thesis.

[77] Bouton C.E., Shaikhouni A., Annetta N.V., Bockbrader M.A., Friedenberg D.A., Nielson D.M., Sharma G., Sederberg P.B., Glenn B.C., Mysiw W.J. (2016). Restoring cortical control of functional movement in a human with quadriplegia. Nature.

[78] Ganzer P.D., Colachis S.C., Schwemmer M.A., Friedenberg D.A., Dunlap C.F., Swiftney C.E., Jacobowitz A.F., Weber D.J., Bockbrader M.A., Sharma G. (2020). Restoring the Sense of Touch Using a Sensorimotor Demultiplexing Neural Interface. Cell.

[79] Ajiboye A.B., Willett F.R., Young D.R., Memberg W.D., Murphy B.A., Miller J.P., Walter B.L., Sweet J.A., Hoyen H.A., Keith M.W. (2017). Restoration of reaching and grasping movements through brain-controlled muscle stimulation in a person with tetraplegia: A proof-of-concept demonstration. Lancet.

[80] Willett F.R., Young D.R., Murphy B.A., Memberg W.D., Blabe C.H., Pandarinath C., Stavisky S.D., Rezaii P., Saab J., Walter B.L. (2019). Principled BCI decoder design and parameter selection using a feedback control model. Sci. Rep..

[81] Schwemmer M.A., Skomrock N.D., Sederberg P.B., Ting J.E., Sharma G., Bockbrader M.A., Friedenberg D.A. (2018). Meeting brain–computer interface user performance expectations using a deep neural network decoding framework. Nat. Med..

[82] Sitaram R., Caria A., Veit R., Gaber T., Rota G., Kuebler A., Birbaumer N. (2007). FMRI brain-computer interface: A tool for neuroscientific research and treatment. Comput. Intell. Neurosci..

[83] Birbaumer N. (2006). Breaking the silence: Brain–computer interfaces (BCI) for communication and motor control. Psychophysiology.

[84] Kim J., Lee J., Han C., Park K. (2019). An Instant Donning Multi-Channel EEG Headset (with Comb-Shaped Dry Electrodes) and BCI Applications. Sensors.

[85] Velasco-Álvarez F., Sancha-Ros S., García-Garaluz E., Fernández-Rodríguez Á., Medina-Juliá M.T., Ron-Angevin R. (2019). UMA-BCI speller: An easily configurable P300 speller tool for end users. Comput. Methods Programs Biomed..

[86] Mowla M.R., Gonzalez-Morales J.D., Rico-Martinez J., Ulichnie D.A., Thompson D.E. (2020). A Comparison of Classification Techniques to Predict Brain-Computer Interfaces Accuracy Using Classifier-Based Latency Estimation. Brain Sci..

[87] Al-Saegh A., Dawwd S.A., Abdul-Jabbar J.M. (2021). Deep learning for motor imagery EEG-based classification: A review. Biomed. Signal Process. Control.

[88] Yoo S.S., Fairneny T., Chen N.K., Choo S.E., Panych L.P., Park H., Lee S.Y., Jolesz F.A. (2004). Brain—Computer interface using fMRI: Spatial navigation by thoughts. Neuroreport.

[89] Montagna F. (2020). Optimized Biosignals Processing Algorithms for New Designs of Human Machine Interfaces on Parallel Ultra-Low Power Architectures. Ph.D. Thesis.

[90] Maymandi H., Perez-Benitez J., Gallegos-Funesa F., Perez-Benitez J. (2020). A Novel Monitor for Practical Brain-Computer Interface Applications Based on Visual Evoked Potential.

[91] Hasan M.A., Khan M.U., Mishra D. (2020). A Computationally Efficient Method for Hybrid EEG-fNIRS BCI Based on the Pearson Correlation. BioMed Res. Int..

[92] Wolpaw J., McFarland D. (1995). Development of an EEG-based brain-computer interface (BCI). Rehabil. Eng. Soc. N. Am..

[93] Flotzinger D., Kalcher J., Wolpaw J. (1993). Off-Line Classification of EEG from the “New York Brain-Computer Interface (BCI)”.

[94] McFarland D., Sarnacki W., Wolpaw J. (1998). EEG-based brain-computer interface (BCI): Multiple selections with one dimensional control. Soc. Neurosci. Abstr..

[95] Pfurtscheller G., Flotzinger D., Kalcher J. (1993). Brain-computer interface—A new communication device for handicapped persons. J. Microcomput. Appl..

[96] Cecotti H. (2011). Spelling with non-invasive Brain–Computer Interfaces–Current and future trends. J. Physiol.-Paris.

[97] DEL R. MILLÁN J., Ferrez P.W., Galán F., Lew E., Chavarriaga R. (2008). Non-invasive brain-machine interaction. Int. J. Pattern Recognit. Artif. Intell..

[98] Tangermann M.W., Krauledat M., Grzeska K., Sagebaum M., Vidaurre C., Blankertz B., Müller K.R. (2008). Playing pinball with non-invasive BCI. Proceedings of the 21st International Conference on Neural Information Processing Systems.

[99] McFarland D.J., Lefkowicz A.T., Wolpaw J.R. (1997). Design and operation of an EEG-based brain-computer interface with digital signal processing technology. Behav. Res. Methods Instrum. Comput..

[100] Miner L.A., McFarland D.J., Wolpaw J.R. (1998). Answering questions with an electroencephalogram-based brain-computer interface. Arch. Phys. Med. Rehabil..

[101] Schalk G., McFarland D.J., Hinterberger T., Birbaumer N., Wolpaw J.R. (2004). BCI2000: A general-purpose brain-computer interface (BCI) system. IEEE Trans. Biomed. Eng..

[102] Millán J.D.R., Renkens F., Mourino J., Gerstner W. Non-invasive brain-actuated control of a mobile robot. Proceedings of the 18th International Joint Conference on Artificial Intelligence.

[103] Kapgate D. (2020). Future of EEG Based Hybrid Visual Brain Computer Interface Systems in Rehabilitation of People with Neurological Disorders. Int. Res. J. Adv. Sci. Hub.

[104] Pfurtscheller G., Neuper C., Guger C., Harkam W., Ramoser H., Schlogl A., Obermaier B., Pregenzer M. (2000). Current trends in Graz brain-computer interface (BCI) research. IEEE Trans. Rehabil. Eng..

[105] Blankertz B., Dornhege G., Krauledat M., Müller K.R., Curio G. (2007). The non-invasive Berlin brain–computer interface: Fast acquisition of effective performance in untrained subjects. NeuroImage.

[106] Vaughan T.M., McFarland D.J., Schalk G., Sarnacki W.A., Krusienski D.J., Sellers E.W., Wolpaw J.R. (2006). The wadsworth BCI research and development program: At home with BCI. IEEE Trans. Neural Syst. Rehabil. Eng..

[107] Pfurtscheller G., Neuper C., Muller G., Obermaier B., Krausz G., Schlogl A., Scherer R., Graimann B., Keinrath C., Skliris D. (2003). Graz-BCI: State of the art and clinical applications. IEEE Trans. Neural Syst. Rehabil. Eng..

[108] del R Millan J., Mouriño J., Franzé M., Cincotti F., Varsta M., Heikkonen J., Babiloni F. (2002). A local neural classifier for the recognition of EEG patterns associated to mental tasks. IEEE Trans. Neural Netw..

[109] Cincotti F., Mattia D., Aloise F., Bufalari S., Schalk G., Oriolo G., Cherubini A., Marciani M.G., Babiloni F. (2008). Non-invasive brain–computer interface system: Towards its application as assistive technology. Brain Res. Bull..

[110] Schembri P., Pelc M., Ma J. (2020). The effect that auditory distractions have on a visual P300 speller while utilizing low-cost off-the-shelf equipment. Computers.

[111] Schembri P., Pelc M., Ma J. The Effect that Auxiliary Taxonomized Auditory Distractions have on a P300 Speller while utilising Low Fidelity Equipment. Proceedings of the 2019 11th Computer Science and Electronic Engineering (CEEC).

[112] Allison B.Z., Kübler A., Jin J. (2020). 30+ years of P300 brain–computer interfaces. Psychophysiology.

[113] Ravi A., Beni N.H., Manuel J., Jiang N. (2020). Comparing user-dependent and user-independent training of CNN for SSVEP BCI. J. Neural Eng..

[114] Li K., Sankar R., Arbel Y., Donchin E. Single trial independent component analysis for P300 BCI system. Proceedings of the 2009 Annual International Conference of the IEEE Engineering in Medicine and Biology Society.

[115] Jin J., Allison B.Z., Kaufmann T., Kübler A., Zhang Y., Wang X., Cichocki A. (2012). The changing face of P300 BCIs: A comparison of stimulus changes in a P300 BCI involving faces, emotion, and movement. PLoS ONE.

[116] Fouad I.A., Labib F.E.Z.M., Mabrouk M.S., Sharawy A.A., Sayed A.Y. (2020). Improving the performance of P300 BCI system using different methods. Netw. Model. Anal. Health Inform. Bioinform..

[117] Eidel M., Kübler A. (2020). Wheelchair Control in a Virtual Environment by Healthy Participants Using a P300-BCI Based on Tactile Stimulation: Training Effects and Usability. Front. Hum. Neurosci..

[118] Liu B., Huang X., Wang Y., Chen X., Gao X. (2020). BETA: A Large Benchmark Database Toward SSVEP-BCI Application. Front. Neurosci..

[119] Chailloux Peguero J.D., Mendoza-Montoya O., Antelis J.M. (2020). Single-Option P300-BCI Performance Is Affected by Visual Stimulation Conditions. Sensors.

[120] Berlad I., Pratt H. (1995). P300 in response to the subject’s own name. Electroencephalogr. Clin. Neurophysiol. Potentials Sect..

[121] Polich J., Margala C. (1997). P300 and probability: Comparison of oddball and single-stimulus paradigms. Int. J. Psychophysiol..

[122] Dutt-Mazumder A., Huggins J.E. (2020). Performance comparison of a non-invasive P300-based BCI mouse to a head-mouse for people with SCI. Brain-Comput. Interfaces.

[123] Cortez S.A., Flores C., Andreu-Perez J. (2020). A Smart Home Control Prototype Using a P300-Based Brain–Computer Interface for Post-stroke Patients. Proceedings of the 5th Brazilian Technology Symposium.

[124] Bulat M., Karpman A., Samokhina A., Panov A. (2020). Playing a P300-BCI VR game based leads to changes in cognitive function of healthy adults. bioRxiv.

[125] Mouli S., Palaniappan R., Molefi E., McLoughlin I. (2020). In-Ear Electrode EEG for Practical SSVEP BCI. Technologies.

[126] Peters B., Bedrick S., Dudy S., Eddy B., Higger M., Kinsella M., McLaughlin D., Memmott T., Oken B., Quivira F. (2020). SSVEP BCI and eye tracking use by individuals with late-stage ALS and visual impairments. Front. Hum. Neurosci..

[127] Hwang H.J., Lim J.H., Jung Y.J., Choi H., Lee S.W., Im C.H. (2012). Development of an SSVEP-based BCI spelling system adopting a QWERTY-style LED keyboard. J. Neurosci. Methods.

[128] Muller-Putz G.R., Pfurtscheller G. (2007). Control of an electrical prosthesis with an SSVEP-based BCI. IEEE Trans. Biomed. Eng..

[129] Horki P., Solis-Escalante T., Neuper C., Müller-Putz G. (2011). Combined motor imagery and SSVEP based BCI control of a 2 DoF artificial upper limb. Med. Biol. Eng. Comput..

[130] Chen X., Zhao B., Wang Y., Gao X. (2019). Combination of high-frequency SSVEP-based BCI and computer vision for controlling a robotic arm. J. Neural Eng..

[131] Lin J.S., Jiang Z.Y. (2015). Implementing remote presence using quadcopter control by a non-invasive BCI device. Comput. Sci. Inf. Technol..

[132] Cho J.H., Jeong J.H., Shim K.H., Kim D.J., Lee S.W. Classification of hand motions within EEG signals for non-invasive BCI-based robot hand control. Proceedings of the 2018 IEEE International Conference on Systems, Man, and Cybernetics (SMC).

[133] Hiremath S.V., Chen W., Wang W., Foldes S., Yang Y., Tyler-Kabara E.C., Collinger J.L., Boninger M.L. (2015). Brain computer interface learning for systems based on electrocorticography and intracortical microelectrode arrays. Front. Integr. Neurosci..

[134] Angelakis E., Hatzis A., Panourias I., Sakas D. (2007). Brain-computer interface: A reciprocal self-regulated neuromodulation. Operative Neuromodulation.

[135] Sorger B., Goebel R. (2020). Real-time fMRI for brain-computer interfacing. Handbook of Clinical Neurology.

[136] Weiskopf N., Mathiak K., Bock S.W., Scharnowski F., Veit R., Grodd W., Goebel R., Birbaumer N. (2004). Principles of a brain-computer interface (BCI) based on real-time functional magnetic resonance imaging (fMRI). IEEE Trans. Biomed. Eng..

[137] Sitaram R., Veit R., Stevens B., Caria A., Gerloff C., Birbaumer N., Hummel F. (2012). Acquired control of ventral premotor cortex activity by feedback training: An exploratory real-time FMRI and TMS study. Neurorehabilit. Neural Repair.

[138] Rota G., Handjaras G., Sitaram R., Birbaumer N., Dogil G. (2011). Reorganization of functional and effective connectivity during real-time fMRI-BCI modulation of prosody processing. Brain Lang..

[139] Sitaram R., Weiskopf N., Caria A., Veit R., Erb M., Birbaumer N. (2007). fMRI brain-computer interfaces. IEEE Signal Process. Mag..

[140] Liberati G., Veit R., Kim S., Birbaumer N., Von Arnim C., Jenner A., Lulé D., Ludolph A.C., Raffone A., Belardinelli M.O. Development of a binary fMRI-BCI for Alzheimer patients: A semantic conditioning paradigm using affective unconditioned stimuli. Proceedings of the 2013 Humaine Association Conference on Affective Computing and Intelligent Interaction.

[141] Simon J., Fishbein P., Zhu L., Roberts M., Martin I. (2020). Functional Magnetic Resonance Imaging-Based Brain Computer Interfaces. Neural Interface Engineering.

[142] Rieke J.D., Matarasso A.K., Yusufali M.M., Ravindran A., Alcantara J., White K.D., Daly J.J. (2020). Development of a Combined, Sequential Real-Time fMRI and fNIRS Neurofeedback System Enhance Motor Learning After Stroke. J. Neurosci. Methods.

[143] Almulla L., Al-Naib I., Althobaiti M. (2020). Hemodynamic responses during standing and sitting activities: A study toward fNIRS-BCI. Biomed. Phys. Eng. Express.

[144] Nazeer H., Naseer N., Khan R.A., Noori F.M., Qureshi N.K., Khan U.S., Khan M.J. (2020). Enhancing classification accuracy of fNIRS-BCI using features acquired from vector-based phase analysis. J. Neural Eng..

[145] Mandal S., Singh B., Thakur K. (2020). Classification of working memory loads using hybrid EEG and fNIRS in machine learning paradigm. Electron. Lett..

[146] Ghonchi H., Fateh M., Abolghasemi V., Ferdowsi S., Rezvani M. Spatio-temporal deep learning for EEG-fNIRS brain computer interface. Proceedings of the 2020 42nd Annual International Conference of the IEEE Engineering in Medicine & Biology Society (EMBC).

[147] Li F., Tao Q., Peng W., Zhang T., Si Y., Zhang Y., Yi C., Biswal B., Yao D., Xu P. (2020). Inter-subject P300 variability relates to the efficiency of brain networks reconfigured from resting-to task-state: Evidence from a simultaneous event-related EEG-fMRI study. NeuroImage.

[148] Martini M.L., Oermann E.K., Opie N.L., Panov F., Oxley T., Yaeger K. (2020). Sensor modalities for brain-computer interface technology: A comprehensive literature review. Neurosurgery.

[149] Jerbi K., Vidal J., Mattout J., Maby E., Lecaignard F., Ossandon T., Hamamé C., Dalal S., Bouet R., Lachaux J.P. (2011). Inferring hand movement kinematics from MEG, EEG and intracranial EEG: From brain-machine interfaces to motor rehabilitation. Irbm.

[150] LaRocco J., Le M.D., Paeng D.G. (2020). A systemic review of available low-cost EEG headsets used for drowsiness detection. Front. Neuroinform..

[151] de Lissa P., Sörensen S., Badcock N., Thie J., McArthur G. (2015). Measuring the face-sensitive N170 with a gaming EEG system: A validation study. J. Neurosci. Methods.

[152] Doudou M., Bouabdallah A., Cherfaoui V. (2018). A Light on Physiological Sensors for Efficient Driver Drowsiness Detection System. Sens. Transducers J..

[153] Aghaei-Lasboo A., Inoyama K., Fogarty A.S., Kuo J., Meador K.J., Walter J.J., Le S.T., Graber K.D., Razavi B., Fisher R.S. (2020). Tripolar concentric EEG electrodes reduce noise. Clin. Neurophysiol..

[154] Liu X., Makeyev O., Besio W. (2020). Improved Spatial Resolution of Electroencephalogram Using Tripolar Concentric Ring Electrode Sensors. J. Sens..

[155] g.tec Medical Engineering | Brain-Computer Interfaces and Neurotechnology. https://www.gtec.at/.

[156] Vasiljevic G.A.M., de Miranda L.C. (2020). Brain–computer interface games based on consumer-grade EEG Devices: A systematic literature review. Int. J. Hum.-Comput. Interact..

[157] Chi Y.M., Wang Y.T., Wang Y., Maier C., Jung T.P., Cauwenberghs G. (2011). Dry and noncontact EEG sensors for mobile brain–computer interfaces. IEEE Trans. Neural Syst. Rehabil. Eng..

[158] Gu X., Cao Z., Jolfaei A., Xu P., Wu D., Jung T.P., Lin C.T. (2020). EEG-based Brain-Computer Interfaces (BCIs): A Survey of Recent Studies on Signal Sensing Technologies and Computational Intelligence Approaches and their Applications. arXiv.

[159] Belkacem A.N., Jamil N., Palmer J.A., Ouhbi S., Chen C. (2020). Brain computer interfaces for improving the quality of life of older adults and elderly patients. Front. Neurosci..

[160] OpenBCI—Open Source Biosensing Tools (EEG, EMG, EKG, and more). https://openbci.com/.

[161] EMOTIV | Brain Data Measuring Hardware and Software Solutions. https://www.emotiv.com/.

[162] Muse™—Meditation Made Easy with the Muse Headband. https://choosemuse.com/.

[163] Stytsenko K., Jablonskis E., Prahm C. (2011). Evaluation of consumer EEG device Emotiv EPOC. MEi: CogSci Conference 2011.

[164] Liu Y., Jiang X., Cao T., Wan F., Mak P.U., Mak P.I., Vai M.I. Implementation of SSVEP based BCI with Emotiv EPOC. Proceedings of the 2012 IEEE International Conference on Virtual Environments Human-Computer Interfaces and Measurement Systems (VECIMS) Proceedings.

[165] EEG—ECG—Biosensors. http://neurosky.com/.

[166] Crowley K., Sliney A., Pitt I., Murphy D. Evaluating a brain-computer interface to categorise human emotional response. Proceedings of the 2010 10th IEEE International Conference on Advanced Learning Technologies.

[167] Lakhan P., Banluesombatkul N., Changniam V., Dhithijaiyratn R., Leelaarporn P., Boonchieng E., Hompoonsup S., Wilaiprasitporn T. (2019). Consumer grade brain sensing for emotion recognition. IEEE Sens. J..

[168] Frey J. Comparison of a consumer grade EEG amplifier with medical grade equipment in BCI applications. Proceedins of the 2016 6th International BCI Meeting – BCI Past, Present and Future, Asilomar Conference Center.

[169] Frey J. (2016). Comparison of an open-hardware electroencephalography amplifier with medical grade device in brain-computer interface applications. arXiv.

[170] Haddix C., Bahrani A.A., Kawala-Janik A., Besio W.G., Yu G., Sunderam S. Trial measurement of movement-related cortical dynamics using electroencephalography and diffuse correlation spectroscopy. Proceedings of the 2017 22nd International Conference on Methods and Models in Automation and Robotics (MMAR).

[171] Makeyev O., Ding Q., Kay S.M., Besio W.G. Sensor integration of multiple tripolar concentric ring electrodes improves pentylenetetrazole-induced seizure onset detection in rats. Proceedings of the 2012 Annual International Conference of the IEEE Engineering in Medicine and Biology Society.

[172] Makeyev O., Ding Q., Martínez-Juárez I.E., Gaitanis J., Kay S.M., Besio W.G. Multiple sensor integration for seizure onset detection in human patients comparing conventional disc versus novel tripolar concentric ring electrodes. Proceedings of the 2013 35th Annual International Conference of the IEEE Engineering in Medicine and Biology Society (EMBC).

[173] Müller-Putz G.R., Scherer R., Pfurtscheller G., Rupp R. (2005). EEG-based neuroprosthesis control: A step towards clinical practice. Neurosci. Lett..

[174] Kuś R., Duszyk A., Milanowski P., Łabęcki M., Bierzyńska M., Radzikowska Z., Michalska M., Żygierewicz J., Suffczyński P., Durka P.J. (2013). On the quantification of SSVEP frequency responses in human EEG in realistic BCI conditions. PLoS ONE.

[175] Volosyak I., Valbuena D., Malechka T., Peuscher J., Gräser A. (2010). Brain–computer interface using water-based electrodes. J. Neural Eng..

[176] Chabuda A., Durka P., Żygierewicz J. (2017). High frequency SSVEP-BCI with hardware stimuli control and phase-synchronized comb filter. IEEE Trans. Neural Syst. Rehabil. Eng..

[177] Tung S.W., Guan C., Ang K.K., Phua K.S., Wang C., Zhao L., Teo W.P., Chew E. Motor imagery BCI for upper limb stroke rehabilitation: An evaluation of the EEG recordings using coherence analysis. Proceedings of the 2013 35th Annual International Conference of the IEEE Engineering in Medicine and Biology Society (EMBC).

[178] Onose G., Grozea C., Anghelescu A., Daia C., Sinescu C., Ciurea A., Spircu T., Mirea A., Andone I., Spânu A. (2012). On the feasibility of using motor imagery EEG-based brain–computer interface in chronic tetraplegics for assistive robotic arm control: A clinical test and long-term post-trial follow-up. Spinal Cord.

[179] Katona J., Kovari A. (2018). The evaluation of bci and pebl-based attention tests. Acta Polytech. Hung..

[180] Fazli S., Danóczy M., Popescu F., Blankertz B., Müller K.R. (2009). Using rest class and control paradigms for brain computer interfacing. International Work-Conference on Artificial Neural Networks.

[181] Bancaud J., Dell M. (1959). Technics and method of stereotaxic functional exploration of the brain structures in man (cortex, subcortex, central gray nuclei). Rev. Neurol..

[182] Herff C., Krusienski D.J., Kubben P. (2020). The Potential of Stereotactic-EEG for Brain-Computer Interfaces: Current Progress and Future Directions. Front. Neurosci..

[183] Guenot M., Isnard J., Ryvlin P., Fischer C., Ostrowsky K., Mauguiere F., Sindou M. (2001). Neurophysiological monitoring for epilepsy surgery: The Talairach SEEG method. Stereotact. Funct. Neurosurg..

[184] Allen P., Fish D., Smith S. (1992). Very high-frequency rhythmic activity during SEEG suppression in frontal lobe epilepsy. Electroencephalogr. Clin. Neurophysiol..

[185] Sharma A., Rai J.K., Tewari R.P. (2020). Scalp electroencephalography (sEEG) based advanced prediction of epileptic seizure time and identification of epileptogenic region. Biomed. Eng. Tech..

[186] Chandrasekaran S., Bickel S., Herrero J.L., Kim J.W., Markowitz N., Espinal E., Bhagat N.A., Ramdeo R., Xu J., Glasser M.F. (2020). Evoking highly focal percepts in the fingertips through targeted stimulation of sulcal regions of the brain for sensory restoration. medRxiv.

[187] Talukdar U., Hazarika S.M., Gan J.Q. (2020). Adaptation of Common Spatial Patterns based on mental fatigue for motor-imagery BCI. Biomed. Signal Process. Control.

[188] Wong C.M., Wang B., Wang Z., Lao K.F., Rosa A., Wan F. (2020). Spatial Filtering in SSVEP-based BCIs: Unified Framework and New Improvements. IEEE Trans. Biomed. Eng..

[189] Gaber A., Ghazali M. (2005). Trends in Brain Computer Interfaces. EURASIP J. Adv. Signal Process..

[190] Zander T.O., Kothe C., Jatzev S., Gaertner M. (2010). Enhancing human-computer interaction with input from active and passive brain-computer interfaces. Brain-Computer Interfaces.

[191] Andreessen L.M., Gerjets P., Meurers D., Zander T.O. (2020). Toward neuroadaptive support technologies for improving digital reading: A passive BCI-based assessment of mental workload imposed by text difficulty and presentation speed during reading. User Model. User-Adapt. Interact..

[192] Elsawy A.S., Eldawlatly S., Taher M., Aly G.M. (2017). MindEdit: A P300-based text editor for mobile devices. Comput. Biol. Med..

[193] Jijun T., Peng Z., Ran X., Lei D. The portable P300 dialing system based on tablet and Emotiv Epoc headset. Proceedings of the 2015 37th Annual International Conference of the IEEE Engineering in Medicine and Biology Society (EMBC).

[194] Tahmasebzadeh A., Bahrani M., Setarehdan S.K. Development of a robust method for an online P300 Speller Brain Computer Interface. Proceedings of the 2013 6th International IEEE/EMBS Conference on Neural Engineering (NER).

[195] Meshriky M.R., Eldawlatly S., Aly G.M. An intermixed color paradigm for P300 spellers: A comparison with gray-scale spellers. Proceedings of the 2017 IEEE 30th International Symposium on Computer-Based Medical Systems (CBMS).

[196] Browarska N., Kawala-Sterniuk A., Zygarlicki J. (2020). Initial study on changes in activity of brain waves during audio stimulation using noninvasive brain—Computer interfaces: Choosing the appropriate filtering method. Bio-Algorithms Med-Syst..

[197] Worthen-Chaudhari L.C., McNally M.P., Deshpande A., Bakaraju V. (2020). In-Home Neurogaming: Demonstrating the impact of valid gesture recognition method on high volume kinematic outcomes. J. Biomech..

[198] Beveridge R., Wilson S., Callaghan M., Coyle D. (2019). Neurogaming with motion-onset visual evoked potentials (mVEPs): Adults versus teenagers. IEEE Trans. Neural Syst. Rehabil. Eng..

[199] Putze F., Vourvopoulos A., Lécuyer A., Krusienski D., i Badia S.B., Mullen T., Herff C. (2020). Brain-Computer Interfaces and Augmented/Virtual Reality. Front. Hum. Neurosci..

[200] Putze F., Weiß D., Vortmann L.M., Schultz T. Augmented Reality Interface for Smart Home Control using SSVEP-BCI and Eye Gaze. Proceedings of the IEEE International Conference on Systems, Man, and Cybernetics.

[201] Juarez D., Tur-Viñes V., Mengual A. (2020). Neuromarketing Applied to Educational Toy Packaging. Front. Psychol..

[202] Nilashi M., Samad S., Ahmadi N., Ahani A., Abumalloh R.A., Asadi S., Abdullah R., Ibrahim O., Yadegaridehkordi E. (2020). Neuromarketing: A Review of Research and Implications for Marketing. J. Soft Comput. Decis. Support Syst..

[203] Hsu L., Chen Y.J. (2019). Music and wine tasting: An experimental neuromarketing study. Br. Food J..

[204] Aldayel M., Ykhlef M., Al-Nafjan A. (2020). Deep Learning for EEG-Based Preference Classification in Neuromarketing. Appl. Sci..

[205] Shahriari M., Feiz D., Zarei A., Kashi E. (2020). The meta-analysis of neuro-marketing studies: Past, present and future. Neuroethics.

[206] Luth T., Ojdanic D., Friman O., Prenzel O., Graser A. Low level control in a semi-autonomous rehabilitation robotic system via a brain-computer interface. Proceedings of the 2007 IEEE 10th International Conference on Rehabilitation Robotics.

[207] Xiong M., Hotter R., Nadin D., Patel J., Tartakovsky S., Wang Y., Patel H., Axon C., Bosiljevac H., Brandenberger A. A Low-Cost, Semi-Autonomous Wheelchair Controlled by Motor Imagery and Jaw Muscle Activation. Proceedings of the 2019 IEEE International Conference on Systems, Man and Cybernetics (SMC).

[208] Stephe S., Kumar T.J.K.V. (2019). Imagery Recognition of EEG Signal Using Cuckoo-Search Masking Empirical Mode Decomposition. Int. J. Innov. Technol. Explor. Eng. (IJITEE).

[209] Zgallai W., Brown J.T., Ibrahim A., Mahmood F., Mohammad K., Khalfan M., Mohammed M., Salem M., Hamood N. Deep learning AI application to an EEG driven BCI smart wheelchair. Proceedings of the 2019 Advances in Science and Engineering Technology International Conferences (ASET).

[210] Rebsamen B., Guan C., Zhang H., Wang C., Teo C., Ang M.H., Burdet E. (2010). A brain controlled wheelchair to navigate in familiar environments. IEEE Trans. Neural Syst. Rehabil. Eng..

[211] Leaman J., La H.M. (2017). A comprehensive review of smart wheelchairs: Past, present, and future. IEEE Trans. Hum.-Mach. Syst..

[212] Murugappan M., Ramachandran N., Sazali Y. (2010). Classification of human emotion from EEG using discrete wavelet transform. J. Biomed. Sci. Eng..

[213] Vortmann L.M., Putze F. Attention-Aware Brain Computer Interface to avoid Distractions in Augmented Reality. Proceedings of the 2020 CHI Conference on Human Factors in Computing Systems.

[214] Browarska N., Kawala-Sterniuk A., Chechelski P., Zygarlicki J. (2020). Analysis of brain waves changes in stressful situations based on horror game with the implementation of virtual reality and brain-computer interface system: A case study. Bio-Algorithms Med-Syst..

[215] Kołodziej M., Tarnowski P., Sawicki D., Majkowski A., Rak R., Bala A., Pluta A. (2020). Fatigue Detection Caused by Office Work with the Use of EOG Signal. IEEE Sens. J..

[216] Wolska A., Sawicki D., Nowak K., Wisełka M., Kołodziej M. Method of Acute Alertness Level Evaluation after Exposure to Blue and Red Light (based on EEG): Technical Aspects. Proceedings of the 6th International Congress on Neurotechnology, Electronics and Informatics (NEUROTECHNIX 2018).

[217] Kubacki A., Jakubowski A. (2018). Controlling the industrial robot model with the hybrid BCI based on EOG and eye tracking. AIP Conference Proceedings.

[218] Garcia A.P., Schjølberg I., Gale S. EEG control of an industrial robot manipulator. Proceedings of the 2013 IEEE 4th International Conference on Cognitive Infocommunications (CogInfoCom).

[219] Mason C., Gadzicki K., Meier M., Ahrens F., Kluss T., Maldonado J., Putze F., Fehr T., Zetzsche C., Herrmann M. From Human to Robot Everyday Activity. Proceedings of the 2020 IEEE/RSJ International Conference on Intelligent Robots and Systems (IROS).

[220] Kosmyna N., Tarpin-Bernard F., Bonnefond N., Rivet B. (2016). Feasibility of BCI control in a realistic smart home environment. Front. Hum. Neurosci..

[221] Saboor A., Rezeika A., Stawicki P., Gembler F., Benda M., Grunenberg T., Volosyak I. (2017). SSVEP-based BCI in a smart home scenario. International Work-Conference on Artificial Neural Networks.

[222] Carabalona R., Grossi F., Tessadri A., Castiglioni P., Caracciolo A., de Munari I. (2012). Light on! Real world evaluation of a P300-based brain–computer interface (BCI) for environment control in a smart home. Ergonomics.

[223] Kim H.J., Lee M.H., Lee M. A BCI based Smart Home System Combined with Event-related Potentials and Speech Imagery Task. Proceedings of the 2020 8th International Winter Conference on Brain-Computer Interface (BCI).

[224] Alrajhi W., Alaloola D., Albarqawi A. Smart home: Toward daily use of BCI-based systems. Proceedings of the 2017 International Conference on Informatics, Health & Technology (ICIHT).

[225] Ang K.K., Guan C. (2015). Brain–computer interface for neurorehabilitation of upper limb after stroke. Proc. IEEE.

[226] Zieliński T.P. (2005). Cyfrowe Przetwarzanie Sygnałów: Od Teorii do Zastosowań.

[227] Miao G.J., Clements M.A. (2002). Digital Signal Processing and Statistical Classification.

[228] Enderle J., Bronzino J. (2012). Introduction to Biomedical Engineering.

[229] Kang H.J., Kawasawa Y.I., Cheng F., Zhu Y., Xu X., Li M., Sousa A.M., Pletikos M., Meyer K.A., Sedmak G. (2011). Spatio-temporal transcriptome of the human brain. Nature.

[230] Kawala-Janik A., Pelc M., Podpora M. (2015). Method for EEG signals pattern recognition in embedded systems. Elektron. Elektrotechnika.

[231] Rodin E., Funke M., Berg P., Matsuo F. (2004). Magnetoencephalographic spikes not detected by conventional electroencephalography. Clin. Neurophysiol..

[232] Wang G., Worrell G., Yang L., Wilke C., He B. (2011). Interictal spike analysis of high-density EEG in patients with partial epilepsy. Clin. Neurophysiol..

[233] Breitling C., Zaehle T., Dannhauer M., Tegelbeckers J., Flechtner H.H., Krauel K. (2020). Comparison between conventional and HD-tDCS of the right inferior frontal gyrus in children and adolescents with ADHD. Clin. Neurophysiol..

[234] Alhaddad M.J. (2012). Common average reference (CAR) improves P300 speller. Int. J. Eng. Technol..

[235] Laiho J. (2020). Recognizing Thoughts from Bioelectric Patterns? A Brain-Computer Interface with Deep Learning. Master’s Thesis.

[236] Wang L., Huang W., Yang Z., Hu X., Zhang C. (2020). A method from offline analysis to online training for the brain-computer interface based on motor imagery and speech imagery. Biomed. Signal Process. Control.

[237] Grozea C., Voinescu C.D., Fazli S. (2011). Bristle-sensors—Low-cost flexible passive dry EEG electrodes for neurofeedback and BCI applications. J. Neural Eng..

[238] Saab J., Battes B., Grosse-Wentrup M., Scherer R., Billinger M., Kreilinger A. (2011). Simultaneous EEG Recordings with Dry and Wet Electrodes in Motor-Imagery.

[239] Klekowicz H. (2012). Opis i Identyfikacja Struktur Przejściowych w Sygnale EEG. Doctoral Thesis.

[240] Kutz M. (2009). Biomedical Engineering and Design Handbook.

[241] Tumanski S. (2006). Principles of Electrical Measurement.

[242] Semmlow J.L., Griffel B. (2014). Biosignal and Medical Image Processing.

[243] Jiang X., Bian G.B., Tian Z. (2019). Removal of artifacts from EEG signals: A review. Sensors.

[244] Chahid A., Laleg-Kirati T.M. (2020). Optimized Biosignals Decomposition and Denoising Using Schrodinger Operator. https://repository.kaust.edu.sa/handle/10754/662791.

[245] Abtahi F., Seoane F., Lindecrantz K. (2014). Electrical bioimpedance spectroscopy in time-variant systems: Is undersampling always a problem?. J. Electr. Bioimpedance.

[246] Causevic E., Morley R.E., Wickerhauser M.V., Jacquin A.E. (2005). Fast wavelet estimation of weak biosignals. IEEE Trans. Biomed. Eng..

[247] Bagchi S., Mitra S.K. (1999). The Nonuniform Discrete Fourier Transform and Its Applications in Signal Processing.

[248] Khan A. (2005). Digital Signal Processing Fundamentals.

[249] Baby Deepa V., Thangaraj P. (2011). A study on classification of EEG Data using the Filters. Int. J. Adv. Comput. Sci. Appl. (IJACSA).

[250] Philips C.L. (2003). Signals, Systems, and Transforms.

[251] Oppenheim A., Willsky A., Young I. (1983). Signals and Systems.

[252] Bruce E.N. (2000). Biomedical Signal Processing and Signal Modeling.

[253] Yang Y., Peng Z., Zhang W., Meng G. (2019). Parameterised time-frequency analysis methods and their engineering applications: A review of recent advances. Mech. Syst. Signal Process..

[254] Kang M., Jung J., Shin S., Kang K.H., Kim Y.T. Multi bio-signal based algorithm using EMD and FFT for stress analysis. Proceedings of the 2020 IEEE International Conference on Consumer Electronics (ICCE).

[255] Xizheng Z., Yin L., Wang W. (2010). Wavelet Time-frequency Analysis of Electro-encephalogram (EEG) Processing. Int. J. Adv. Comput. Sci. Appl..

[256] Geetha G., Geethalakshmi S. (2011). Scrutinizing different techniques for artifact removal from EEG signals. Int. J. Eng. Sci. Technol. (IJEST).

[257] George S.T., Subathra M., Sairamya N., Susmitha L., Premkumar M.J. (2020). Classification of epileptic EEG signals using PSO based artificial neural network and tunable-Q wavelet transform. Biocybern. Biomed. Eng..

[258] Teolis A. (1998). Computational Signal Processing with Wavelets.

[259] Kawala A., Khoma V., Zmarzły D., Sovyn Y. (2008). Use of wavelet transform for qualification of rheograms characteristic points. Przegląd Elektrotechniczny.

[260] Nishad A., Upadhyay A., Reddy G.R.S., Bajaj V. (2020). Classification of epileptic EEG signals using sparse spectrum based empirical wavelet transform. Electron. Lett..

[261] Desai R., Porob P., Rebelo P., Edla D.R., Bablani A. (2020). EEG Data Classification for Mental State Analysis Using Wavelet Packet Transform and Gaussian Process Classifier. Wirel. Pers. Commun..

[262] Moghavvemi M., Attaran A., Esfahani M.M. (2011). EEG artifact signals tracking and filtering in real time for command control application. 5th Kuala Lumpur International Conference on Biomedical Engineering 2011.

[263] Supratak A., Wu C., Dong H., Sun K., Guo Y. (2016). Survey on feature extraction and applications of biosignals. Machine Learning for Health Informatics.

[264] Maćkiewicz A., Ratajczak W. (1993). Principal components analysis (PCA). Comput. Geosci..

[265] Richardson M. Principal Component Analysis. http://people.maths.ox.ac.uk/richardsonm/SignalProcPCA.pdf.

[266] Molla M.K.I., Islam M.R., Tanaka T., Rutkowski T.M. (2012). Artifact suppression from EEG signals using data adaptive time domain filtering. Neurocomputing.

[267] Domínguez-Jiménez J.A., Campo-Landines K.C., Martínez-Santos J., Delahoz E.J., Contreras-Ortiz S. (2020). A machine learning model for emotion recognition from physiological signals. Biomed. Signal Process. Control.

[268] Elkerdawy M., Elhalaby M., Hassan A., Maher M., Shawky D., Badawi A. Building Cognitive Profiles of Learners Using EEG. Proceedings of the 2020 11th International Conference on Information and Communication Systems (ICICS).

[269] Martinek R., Kahankova R., Jezewski J., Jaros R., Mohylova J., Fajkus M., Nedoma J., Janku P., Nazeran H. (2018). Comparative effectiveness of ICA and PCA in extraction of fetal ECG from abdominal signals: Toward non-invasive fetal monitoring. Front. Physiol..

[270] Jobst B.C., Bartolomei F., Diehl B., Frauscher B., Kahane P., Minotti L., Sharan A., Tardy N., Worrell G., Gotman J. (2020). Intracranial EEG in the 21st Century. Epilepsy Curr..

[271] Reza M.S., Ma J. ICA and PCA integrated feature extraction for classification. Proceedings of the 2016 IEEE 13th International Conference on Signal Processing (ICSP).

[272] Pająk M., Muślewski Ł., Landowski B., Grządziela A. (2019). Fuzzy identification of the reliability state of the mine detecting ship propulsion system. Pol. Marit. Res..

[273] Zadeh L.A. (1988). Fuzzy logic. Computer.

[274] Al-Kadi D., Muhiuddin G. (2020). Bipolar fuzzy BCI-implicative ideals of BCI-algebras. Ann. Commun. Math..

[275] Ghumman M.K., Singh S., Singh N., Jindal B. (2020). Optimization of parameters for improving the performance of EEG-based BCI system. J. Reliab. Intell. Environ..

[276] Abbasi H., Bennet L., Gunn A.J., Unsworth C.P. (2017). Robust wavelet stabilized ‘footprints of Uncertainty’ for fuzzy system classifiers to automatically detect sharp waves in the EEG after hypoxia ischemia. Int. J. Neural Syst..

[277] Plerou A., Vlamou E., Papadopoulos V. (2016). EEG Signal Pattern Recognition Analysis: Fuzzy Logic Systems Ascendancy. Adv. Fuzzy Sets Syst..

[278] Krishnamurthi R., Goyal M. (2019). Hybrid Neuro-fuzzy Method for Data Analysis of Brain Activity Using EEG Signals. Soft Computing and Signal Processing.

[279] Jiang Y., Gu X., Ji D., Qian P., Xue J., Zhang Y., Zhu J., Xia K., Wang S. (2020). Smart Diagnosis: A Multiple-Source Transfer TSK Fuzzy System for EEG Seizure Identification. ACM Trans. Multimed. Comput. Commun. Appl. (TOMM).

[280] Zadeh L.A. (2008). Is there a need for fuzzy logic?. Inf. Sci..

[281] Osowski S., Cichocki A., Siwek K. (2006). MATLAB w Zastosowaniu do Obliczeń Obwodowych i Przetwarzania Sygnałów.

[282] Kawala-Janik A., Bauer W., Żołubak M., Baranowski J. (2016). Early-stage pilot study on using fractional-order calculus-based filtering for the purpose of analysis of electroencephalography signals. Stud. Log. Gramm. Rhetor..

[283] Kawala-Janik A., Bauer W., Al-Bakri A., Haddix C., Yuvaraj R., Cichon K., Podraza W. (2017). Implementation of Low-Pass Fractional Filtering for the Purpose of Analysis of Electroencephalographic Signals. Proceedings of the Non-Integer Order Calculus and its Applications: 9th International Conference on Non-Integer Order Calculus and Its Applications.

[284] Bauer W., Kawala-Janik A. (2017). Implementation of bi-fractional filtering on the arduino uno hardware platform. Theory and Applications of Non-Integer Order Systems.

[285] Baranowski J., Bauer W., Zagórowska M., Piątek P. (2016). On digital realizations of non-integer order filters. Circuits Syst. Signal Process..

[286] Popović N.B., Miljković N., Šekara T.B. Electrogastrogram and electrocardiogram interference: Application of fractional order calculus and Savitzky-Golay filter for biosignals segregation. Proceedings of the 2020 19th International Symposium INFOTEH-JAHORINA (INFOTEH).

[287] Baranowski J., Piątek P., Kawala-Janik A., Zagórowska M., Bauer W., Dziwiński T. (2015). Non-integer order filtration of electromyographic signals. Advances in Modelling and Control of Non-Integer-Order Systems.

[288] Awal M.A., Mostafa S.S., Ahmad M. (2011). Performance analysis of Savitzky-Golay smoothing filter using ECG signal. Int. J. Comput. Inf. Technol..

[289] Trinh P.T., Brossier R., Métivier L., Virieux J., Wellington P. (2017). Bessel smoothing filter for spectral-element mesh. Geophys. J. Int..

[290] Luo J., Ying K., He P., Bai J. (2005). Properties of Savitzky–Golay digital differentiators. Digit. Signal Process..

[291] Savitzky A., Golay M.J. (1964). Smoothing and differentiation of data by simplified least squares procedures. Anal. Chem..

[292] Guiñón J.L., Ortega E., García-Antón J., Pérez-Herranz V. Moving average and Savitzki-Golay smoothing filters using Mathcad. Proceedings of the 2007 International Conference on Engineering Education – ICEE 2007.

[293] Grzechca D., Szczeponik A. (2020). Comparison of Filtering Methods for Enhanced Reliability of a Train Axle Counter System. Sensors.

[294] Choi G.H., Moon H.M., Pan S.B. (2017). Biometrics system technology trends based on biosignal. J. Digit. Converg..

[295] Tsoi A.C., So D., Sergejew A. (1994). Classification of electroencephalogram using artificial neural networks. Adv. Neural Inf. Process. Syst..

[296] Ko W., Jeon E., Jeong S., Suk H.I. (2020). Multi-Scale Neural network for EEG Representation Learning in BCI. arXiv.

[297] Joseph A.F.A., Govindaraju C. (2021). Minimizing electrodes for effective brain computer interface. Biomed. Signal Process. Control.

[298] Szczęsna A., Błaszczyszyn M., Kawala A. (2020). Convolutional neural network in upper limb functional motion analysis after stroke. PeerJ.

[299] Garcia-Moreno F.M., Bermudez-Edo M., Rodríguez-Fórtiz M.J., Garrido J.L. A CNN-LSTM Deep Learning Classifier for Motor Imagery EEG Detection Using a Low-invasive and Low-Cost BCI Headband. Proceedings of the 2020 16th International Conference on Intelligent Environments (IE).

[300] Sussillo D., Nuyujukian P., Fan J.M., Kao J.C., Stavisky S.D., Ryu S., Shenoy K. (2012). A recurrent neural network for closed-loop intracortical brain–machine interface decoders. J. Neural Eng..

[301] Sussillo D., Stavisky S.D., Kao J.C., Ryu S.I., Shenoy K.V. (2016). Making brain–machine interfaces robust to future neural variability. Nat. Commun..

[302] Skomrock N.D., Schwemmer M.A., Ting J.E., Trivedi H.R., Sharma G., Bockbrader M.A., Friedenberg D.A. (2018). A Characterization of Brain-Computer Interface Performance Trade-Offs Using Support Vector Machines and Deep Neural Networks to Decode Movement Intent. Front. Neurosci..

[303] Chaudhary U., Birbaumer N., Ramos-Murguialday A. (2016). Brain–computer interfaces for communication and rehabilitation. Nat. Rev. Neurol..

[304] Sebastián-Romagosa M., Cho W., Ortner R., Murovec N., Von Oertzen T., Kamada K., Allison B.Z., Guger C. (2020). Brain Computer Interface Treatment for Motor Rehabilitation of Upper Extremity of Stroke Patients—A Feasibility Study. Front. Neurosci..

[305] Mak J.N., Wolpaw J.R. (2009). Clinical applications of brain-computer interfaces: Current state and future prospects. IEEE Rev. Biomed. Eng..

[306] Mowla M.R., Cano R.I., Dhuyvetter K.J., Thompson D.E. (2020). Affective Brain-Computer Interfaces: Choosing a Meaningful Performance Measuring Metric. Comput. Biol. Med..

[307] Sawangjai P., Hompoonsup S., Leelaarporn P., Kongwudhikunakorn S., Wilaiprasitporn T. (2019). Consumer grade eeg measuring sensors as research tools: A review. IEEE Sens. J..

[308] Wierzgała P., Zapała D., Wojcik G.M., Masiak J. (2018). Most popular signal processing methods in motor-imagery BCI: A review and meta-analysis. Front. Neuroinform..

[309] Grübler G., Al-Khodairy A., Leeb R., Pisotta I., Riccio A., Rohm M., Hildt E. (2014). Psychosocial and ethical aspects in non-invasive EEG-based BCI research—a survey among BCI users and BCI professionals. Neuroethics.

[310] Schermer M. (2009). The mind and the machine. On the conceptual and moral implications of brain-machine interaction. Nanoethics.

[311] Thinnes-Elker F., Iljina O., Apostolides J.K., Kraemer F., Schulze-Bonhage A., Aertsen A., Ball T. (2012). Intention concepts and brain-machine interfacing. Front. Psychol..

